# Identification, synthesis and mass spectrometry of a macrolide from the African reed frog *Hyperolius cinnamomeoventris*

**DOI:** 10.3762/bjoc.12.269

**Published:** 2016-12-13

**Authors:** Markus Menke, Pardha Saradhi Peram, Iris Starnberger, Walter Hödl, Gregory FM Jongsma, David C Blackburn, Mark-Oliver Rödel, Miguel Vences, Stefan Schulz

**Affiliations:** 1 Technische Universität Braunschweig, Institute of Organic Chemistry, Hagenring 30, 38106 Braunschweig, Germany; 2 Department for Integrative Zoology, Althanstraße 14, 1090 Vienna, Austria; 3 Florida Museum of Natural History, University of Florida, Gainesville, Florida 32611, United States of America; 4 Museum für Naturkunde, Leibniz Institute for Evolution and Biodiversity Science, Invalidenstr. 43, 10115 Berlin, Germany; 5 Technische Universität Braunschweig, Institute of Zoology, 38106 Braunschweig, Germany

**Keywords:** chemical communication, chiral gas chromatography, macrocyclic lactones, ring-closing metathesis, pheromones

## Abstract

The contents of the gular glands of the male African reed frog *Hyperolius cinnamomeoventris* consist of a mixture of aliphatic macrolides and sesquiterpenes. While the known macrolide gephyromantolide A was readily identified, the structure of another major component was suggested to be a tetradecen-13-olide. The synthesis of the two candidate compounds (Z)-5- and (Z)-9-tetradecen-13-olide revealed the former to be the naturally occurring compound. The synthesis used ring-closing metathesis as key step. While the Hoveyda–Grubbs catalyst furnished a broad range of isomeric products, the (*Z*)-selective Grubbs catalyst lead to pure (*Z*)-products. Analysis by chiral GC revealed the natural frog compound to be (5*Z*,13*S*)-5-tetradecen-13-olide (**1**). This compound is also present in the secretion of other hyperoliid frogs as well as in femoral glands of male mantellid frogs such as *Spinomantis aglavei*. The mass spectra of the synthesized macrolides as well as their rearranged isomers obtained during ring-closing metathesis showed that it is possible to assign the location of the double bond in an unsaturated macrolide on the basis of its EI mass spectrum. The occurrence of characteristic ions can be explained by the fragmentation pathway proposed in the article. In contrast, the localization of a double bond in many aliphatic open-chain compounds like alkenes, alcohols or acetates, important structural classes of pheromones, is usually not possible from an EI mass spectrum. In the article, we present the synthesis and for the first time elucidate the structure of macrolides from the frog family Hyperoliidae.

## Introduction

The lactone motif is found in many compounds that are used in chemical communication. Among them, macrocyclic lactones are an important class because of their biosynthetic availability and their inherent compound properties. During the biosynthesis of macrocyclic lactones, a fatty acid precursor is often oxidized near the end of the chain to form a polar hydroxy acid. The following ring-closure reduces the hydrophilicity of the compound and increases its vapor pressure, making the resulting macrocycle well-suited to serve as a signal [1]. Fatty acid derived macrolactones were therefore repeatedly invented during evolution and are used by different animals such as bees, beetles, butterflies, cockroaches, or frogs as pheromones [1].

**Figure 1:**
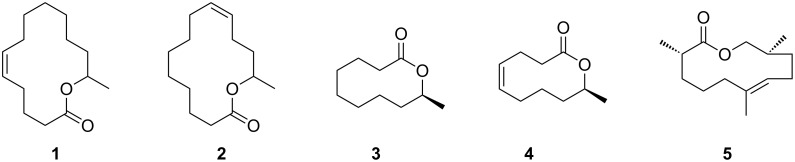
Macrolactones produced in scent glands of frogs: (*Z*)-Tetradec-5-en-13-olide (**1**) or (*Z*)-tetradec-9-en-13-olide (**2**), phoracantolide I (**3**), phoracantolide J (**4**), gephyromantolide A (**5**).

Finding and choosing mates in frogs is usually regarded as being primarily acoustically mediated. Nevertheless, some families like the Mantellidae from Madagascar also use chemical cues. Macrocyclic lactones such as phoracantholide I (**3**), phoracantholide J (**4**) or gephyromantolide A (**5**), are released by males from femoral glands to serve as signals, often accompanied by secondary alcohols (Figure 1) [2–4]. Another frog family most likely using volatile compounds during courtship are the African reed frogs, Hyperoliidae. The males of most species emit acoustic cues to attract females. During the call, an often conspicuously colored gland on their vocal sac (the gular gland), only innervated during the mating season in at least some species, releases a complex blend of volatiles when exposed [5]. A first analysis revealed the presence of mostly unknown terpenes, macrolides and other components in species specific compositions [5].

Detailed exploration of the function of these frog volatiles requires the analysis of the secretion and the synthesis of the identified compounds for biological testing. Because of the small amount of natural material available, only GC–MS investigations can be used to identify the compounds. The analysis revealed unsaturated macrocyclic lactones to be major constituents of the secretion of the cinnamon-bellied reed frog, *Hyperolius cinnamomeoventris*. The location and configuration of the double bond might be relevant for their function, as it is the case in many insect pheromones. In typical pheromone components of insects, including long chain alkenes, alkenols, or unsaturated aldehydes, the location of the double bond can usually not be determined by analysis of their mass spectra. Positional isomers often exhibit almost identical spectra [6] and derivatization or special mass spectrometric techniques are needed to localize double bonds [7–9]. Because this might also be the case for macrolides [10], it is of interest for their identification to compare mass spectra of positional isomers of unsaturated macrolactones. Furthermore, a fast synthetic strategy is needed to synthesize various isomers. In the present work we describe 1) the identification of macrolides from *H. cinnamomeoventris*, 2) discuss EI mass spectra of unsaturated macrolactones, and 3) show how ring-closing metathesis reaction conditions can be selected to obtain either pure compounds or a library of compounds useful for evaluation of their mass spectra.

## Results and Discussion

**Figure 2:**
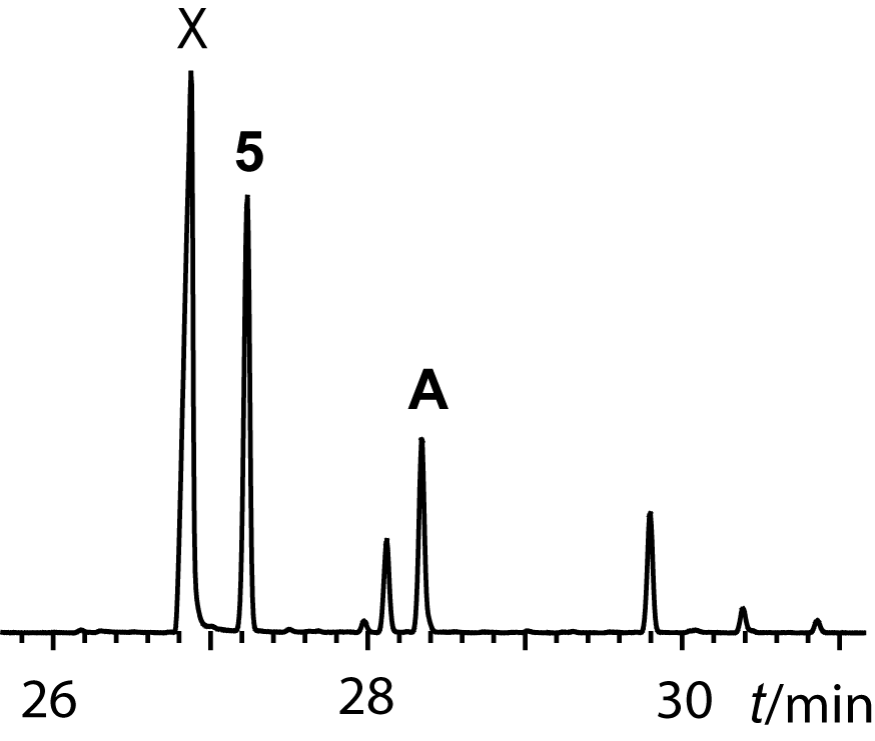
Total ion chromatogram of the gular gland extract of *Hyperolius cinnamomeoventris*. X: frog anaesthetic ethyl 3-aminobenzoate.

The GC–MS analysis of a gular gland extract of *Hyperolius cinnamomeoventris* showed the presence of several unknown compounds, sesquiterpenes and macrolides (Figure 2).

Gephyromantolide A (**5**), known from the mantellid frog *Gephyromantis boulengeri* [4], was readily identified. A second macrolide (**A** in Figure 2) showed a mass spectrum similar to that reported for (*Z*)-tetradec-5-en-13-olide (**1**) by Millar et al. [11], exhibiting a molecular ion at *m/z* 224. This macrolide, called cucujolide III, is used by the flat grain beetle *Cryptolestes pusillus* as pure (*S*)-enantiomer and by *C. turcicus* as a 33:67 *R*/*S* mixture [12], and acts as a synergist to the respective pheromones [11,13]. Microhydrogenation of the frog extract furnished 13-tetradecanolide, thus confirming compound **A** to be a 13-tetradecenolide. The double bond position in this macrolide likely is at C-5, because of the similarity of the mass spectrum to that of the beetle macrolide. Nevertheless, because locating double bonds in such compounds based on the mass spectrum alone seemed not to be reliable, we opted to synthesize two positional isomers.

Compound **1** is biosynthetically formed by the *Cryptolestes* beetles starting from oleic acid [14] that is shortened to 5-tetradecenoic acid, followed by ω − 1 oxidation and ring closure [15]. Another possibility would be that a common saturated acid such as stearic acid is chain-shortened first to tetradecanoic acid, on which a common Δ9-desaturase is acting, leading after ω − 1 oxidation and ring closure to tetradec-9-en-13-olide (**2**). Therefore, we opted to synthesize **2** as well.

To allow later enantiomer determination of **A**, an enantioselective synthetic strategy was followed. Several synthetic routes for the synthesis of **1** have been reported [16–20]. These syntheses were performed before the advent of ring-closing metathesis (RCM), requiring more than 10 steps each. RCM can shorten the synthesis remarkably, but requires careful selection of the RCM catalyst to control the double bond configuration. For example, Fürstner and Langemann obtained *rac*-**1** in 31:69 (*E*/*Z*)-mixture using a Ru-carbene type catalyst similar to a Grubbs I catalyst [21].

**Scheme 1:**
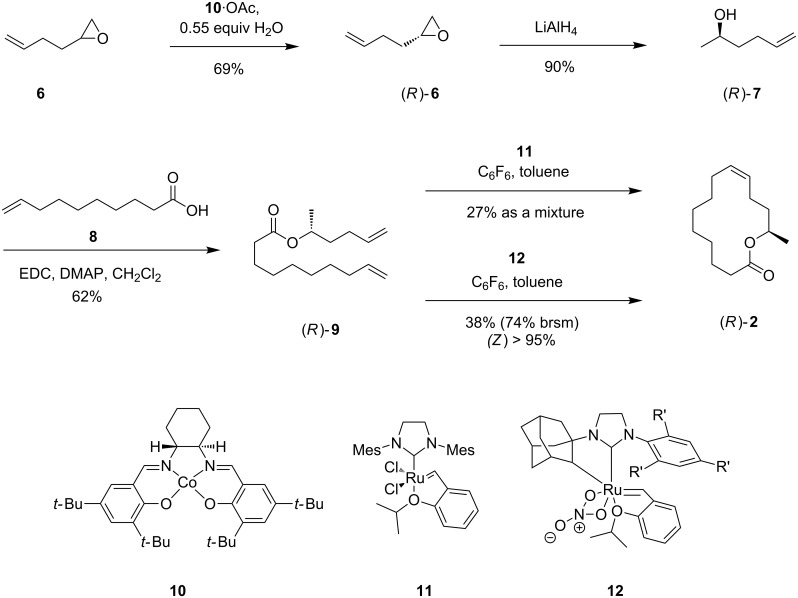
Synthesis of (9*Z*,13*R*)-tetradec-9-en-13-olide (**2**).

The synthesis of (*R*)-**2** using RCM as key step is shown in Scheme 1. Enantiomerically pure 1,2-epoxyhex-5-ene (**6**) was obtained by Jacobsen hydrolytic kinetic resolution on commercially available **6** (Scheme 1) [22–23]. Surprisingly, the yield of 69% of the (*R*)-enantiomer was higher than the theoretically upper limit of 50%. We discovered that **6**, sold by Acros Organics as racemic compound, was actually enriched in the desired (*R*)-enantiomer (see Supporting Information File 1 for optical rotation values). Diene (*R*)-**9** was obtained by reduction of the epoxide **6** with LiAlH_4_ to form alcohol (*R*)-**7**, followed by esterification with 9-decenoic acid (**8**) using 1-ethyl-3-(3-dimethylaminopropyl)carbodiimide (EDC) and 4-(dimethylamino)pyridine (DMAP) [24–25]. The following RCM was performed using Grubbs–Hoveyda II catalyst (**11**) and hexafluorobenzene as an additive [26]. During the reaction isomerization occurred, leading to a mixture of positional isomers and chain shortened as well as elongated products. Addition of *p*-benzoquinone [27] and improved purification methods [28] suppressed isomer formation only partially. Replacing catalyst **11** with the Grubbs second generation catalyst (1,3-bis(2,4,6-trimethylphenyl)-2-imidazolidinylidene)dichloro(phenylmethylene)(tricyclohexylphosphine)ruthenium reduced the isomerization, leading to an (*E*/*Z*)-mixture of **2**. Finally, the (*Z*)-selective Grubbs catalyst **12** furnished the best results [29–30]. This catalyst yielded only the desired product (*R*)-**2** with a (*Z*)-configured double bond, although in moderate yield (see Supporting Information File 1 for full experimental data).

**Scheme 2:**
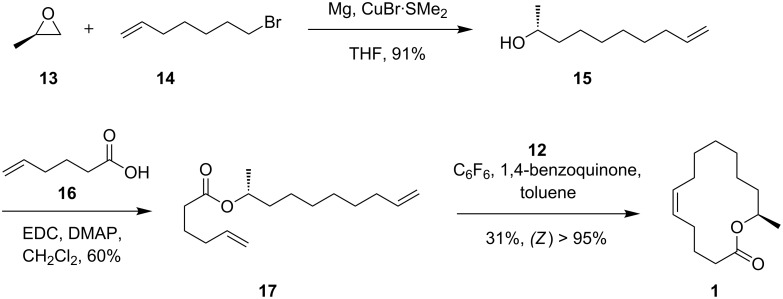
Synthesis of (5*Z*,13*R*)-tetradec-5-en-13-olide ((*R*)-**1**). The enantiomer was obtained in a similar sequence, starting from (*S*)-propylene oxide instead of **13**.

**Figure 3:**
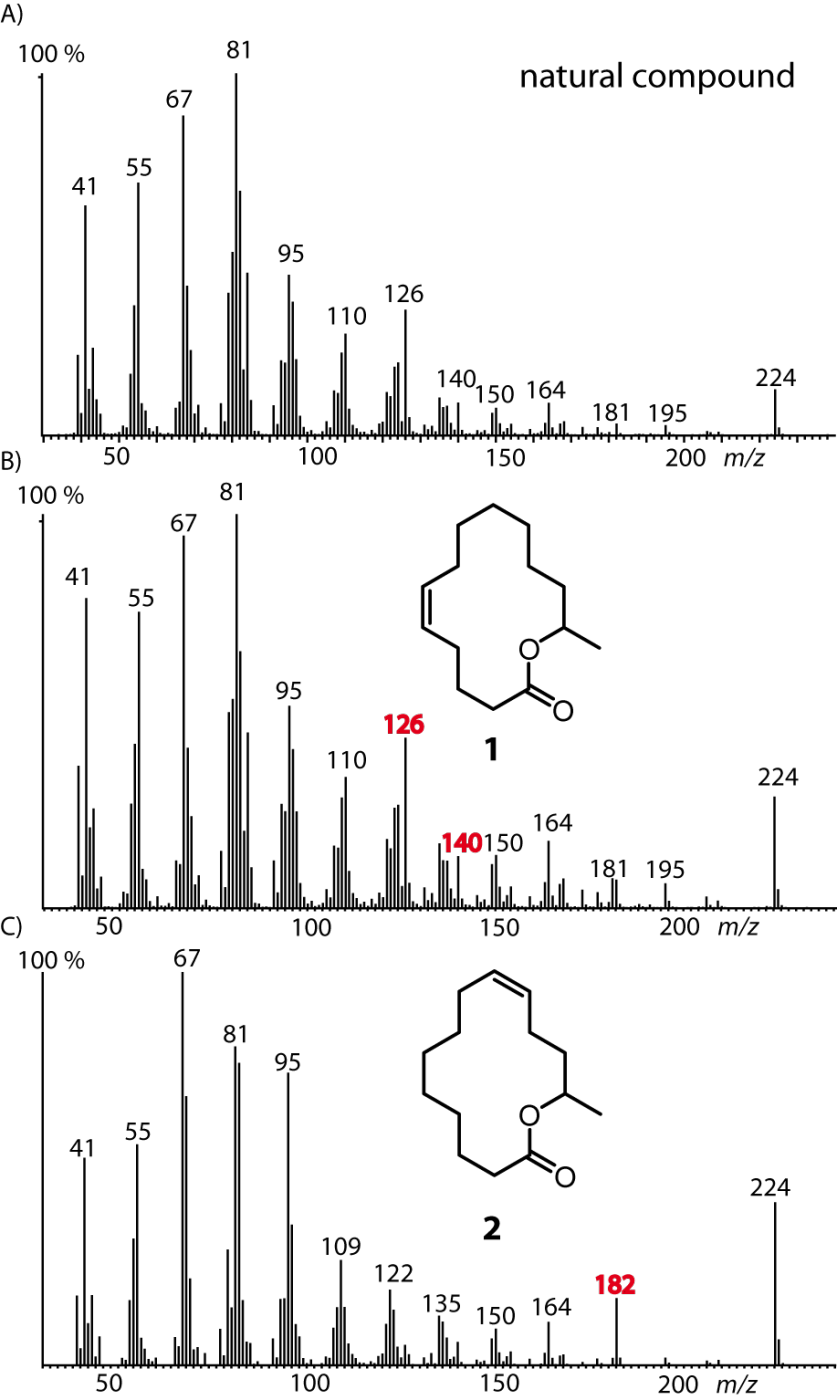
Mass spectra of A) the natural compound **A**, B) (*Z*)-tetradec-5-en-13-olide (**1**), and C) (*Z*)*-*tetradec-9-en-13-olide (**2**). Characteristic ions useful for location of the double bond are marked in red.

A similar synthetic strategy was applied for the synthesis of macrolide **1** (Scheme 2). The stereogenic center was introduced using commercially available (*R*)-propylene oxide (**13**) as starting material. After copper-catalyzed opening of the epoxide with 6-heptenylmagnesium bromide obtained from 7-bromo-1-heptene (**14**) and Steglich esterification with 5-hexenoic acid (**16**), RCM using (*Z*)-selective Grubbs catalyst **12** was used to synthesize macrolide (*R*)-**1** without any isomerization. Comparison of the mass spectra (Figure 3) and gas chromatographic retention times of pure (*Z*)-**1**, the (*E*/*Z*)-mixture obtained by the Hoyveda–Grubbs II catalyst, and **2** with those of the natural compound proved the frog compound to be (*Z*)-tetradec-5-en-13-olide (**1**).

**Figure 4:**
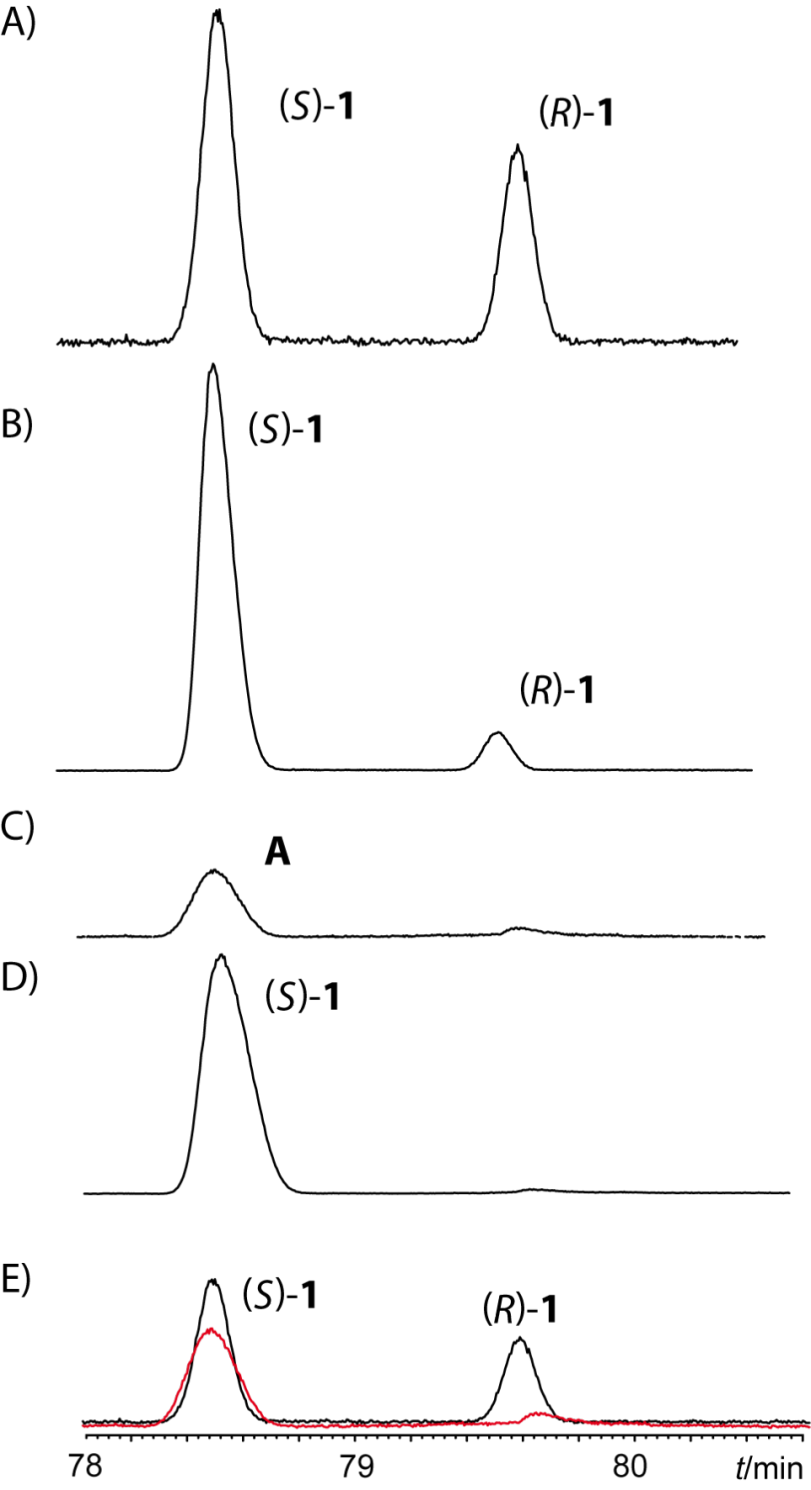
Total ion chromatogram of the enantiomer separation of (*Z*)-**1** on a chiral β-TBDMS- Hydrodex phase. Temperature program: isothermal for 60 min at 110 °C, then with 2 °C/min to 160 °C, followed by a sharp ramp with 25 °C/min to 220 °C. A) Racemic mixture, B) coinjection of racemic mixture and (*S*)-**1**, C) natural extract, D) coinjection of the natural extract and (*S*)-**1**, E) Overlay of A and C showing that the minor peak present in the extract is not the (*R*)-enantiomer of **1**. Black: racemate, red: natural extract. Peak identities were confirmed by mass spectrometry.

For the determination of the absolute configuration of the natural compound, the other enantiomer (*S*)-**1** was needed as well. It was synthesized according to the synthesis shown in Scheme 2, starting from (*S*)-propylene oxide instead of the (*R*)-enantiomer **13**. The stereochemistry was determined by chiral gas chromatography as shown in Figure 4. The coinjection of pure (*S*)-**1** with the racemic mixture proved the first eluting peak to be this enantiomer. Injection of the natural sample as well as (*S*)-**1** showed both compounds to be enantiomerically pure. Finally, coinjection of both samples showed their identity.

With the synthetic material in hand, we analyzed the EI mass spectra of **1** and **2**. No significant differences were detectable between the *E*/*Z*-isomers of each compound. Nevertheless, contrary to open chain compounds, characteristic differences could be found for the positional isomers. While most ions are similar in both spectra, the prominent ion *m/z* 126 present in **1** is shifted to *m/z* 182 in **2**. These ions can be used to assign the location of the double bond in the macrocyclic ring.

**Figure 5:**
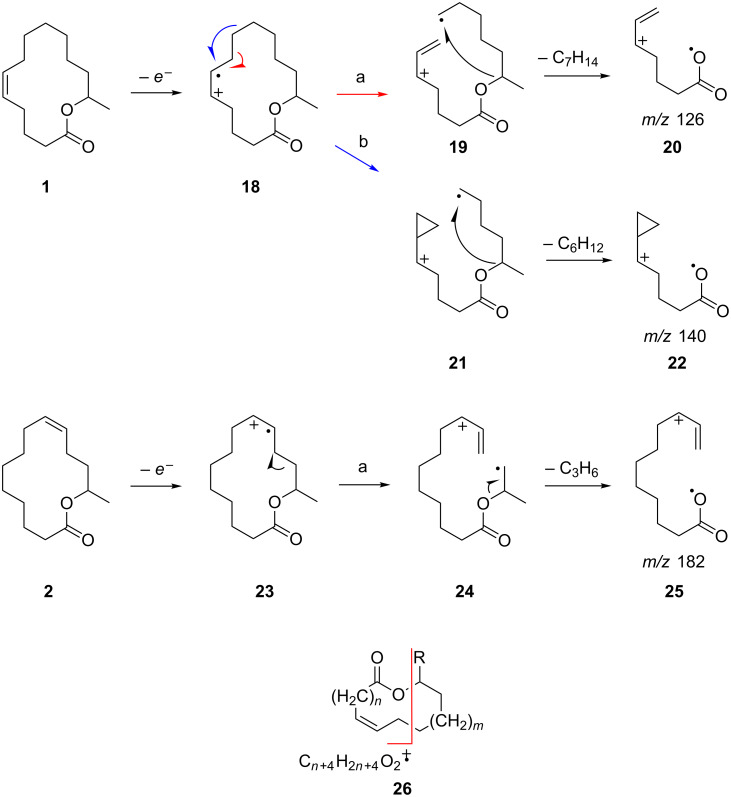
Proposed mass spectrometric fragmentation of macrolides **1** and **2** leading to diagnostic ions of the ion series C*_n_*H_2_*_n_*_−4_O_2_ (C*_n_*_+4_H_2_*_n_*_+4_O_2_ in the figure), indicating the position of the double bond in the ring.

A possible fragmentation pathway leading to these ions is shown in Figure 5. One can speculate that after ionization of the double bond in **1** an allylic cleavage occurs, leading to the radical cation **18** along pathway a. An additional bond cleavage of a C–O single bond releases a neutral molecule, e.g., methylcyclohexane, giving rise to ion *m/z* 126 (**20**). High-resolution mass spectral data support the hypothesis because the ions *m/z* 126 as well as *m/z* 182 (**25**) formed from **2** have a molecular composition of C_7_H_10_O_2_ (HRMS: 126.07005 found, calcd. 126.0681) or C_11_H_18_O_2_ (HRMS: found 182.13206, calcd. 182.1307), respectively. Ion **20** is accompanied by the ion *m/z* 140 (HRMS: found 140.08545, calcd. 140.08373) of the same ion series C*_n_*H_2_*_n_*_–4_O_2_ that occurs in lower abundance. Its formation along pathway b can be explained by homoallylic cleavage of **18** into **21** and further fragmentation into **22**. The formation of the ions of this series is obviously determined by the position of the double bond in the chain. Therefore, ion *m/z* 182 of **2** arises by the same mechanism, indicating the location of the double bond at C-9 (Figure 5). A b-type ion cannot be formed, because pathway b is not operative due to the close proximity of the C–O group. The pathways shown in Figure 5 are speculative. Another possible mechanisms leading to the same ions and starting with ionization of the C–O oxygen atom instead of the double bond is shown in Supporting Information File 1 (see Figure S1). Nevertheless, the proposal is useful to predict the position of double bonds in mono-unsaturated macrolides. This position can be deduced from the ion C*_n_*_+4_H_2_*_n_*_+4_O_2,_ generated by a double bond in the *n* + 2 position, as shown in **26**.

We subsequently tested our method with mass spectra of other macrolides previously reported or present in our compound library. In the mass spectra of (*Z*)-octadec-9-en-13-olide [31] and octadec-9-en-17-olide peaks at *m/z* 182 with higher and *m/z* 196 with lower intensity are present, consistent with a C-9 double bond. In contrast, (*Z*)-octadec-11-en-13-olide does not show such ions, because the close proximity of the double bond and the C–O group does not allow fragmentation along pathway a or b. The spectra of 4-methyl-5-decen-9-olide and 8-methyl-5-decen-9-olide [3] exhibit the expected ions *m/z* 140 and *m/z* 126, respectively. Pathway b is not operative because of the proximity of the C–O group. A slight alteration of the fragmentation can be observed if the double bond is located at C-4, as in phoracantholide J, 4-decen-9-olide [32]. Now in pathway a, additionally an H-atom is transferred leading to the uneven ion C*_n_*H_2_*_n_*_−3_O_2_, *m*/*z* 113, while pathway b remains unchanged, furnishing *m*/*z* 126. In the methylated analog 6-methyl-4-decen-9-olide [3], these ions shift to *m*/*z* 127 and 140. If the double bond moves closer to the C=O group as in 3-dodecen-11-olide [33], both ions a and b are visible, but their abundance is so low that their diagnostic value is largely decreased. Another limitation is that mass spectra of unsaturated unbranched macrolactones, formally derived from ω-hydroxy acids, do not show the characteristic ions generated by pathways a or b. Obviously, the primary C–O bond is not attacked by intermediates like **19**. Nevertheless, the mass spectrometric interpretation presented here will largely ease the identification of a broad range of unsaturated macrolides. The procedure may also be applicable for di- or triunsaturated compounds.

We then focused on the products of the RCM using the Hoveyda–Grubbs II catalyst **11**. It is well known that during RCM isomerization of the double bond might occur, especially with the Grubbs second generation catalysts, leading to products with migrated double bonds or smaller ring size [27,34–36]. Both types of products, 11-dodecenolides, 12-tridecenolides and isomerized 13-tetradecenolides were obtained. In addition, ring expanded 14-pentadecenolides were observed. The latter can be explained by dimerization of esters **9** or **17**, isomerization of the double bond, and final ring closure, leading to either ring-contracted or expanded products. Such processes have been reported before in polymerization experiments using Grubbs catalysts [36–38]. While the isomerization is usually regarded as a negative side reaction in RCM, it turned out to be advantageous for the identification of naturally occurring macrolides in our hands. The isomerized mixtures constitute a library of closely related macrolides differing slightly in position of double bonds and ring size. With the diagnostic mass spectrometric ions discussed above, each compound can be assigned a structure after GC–MS analysis. These mass spectra together with the gas chromatographic retention index are stored in our EIMS database, easing identification of similar compounds in the future. Using this approach, we could collect mass spectra of 5-dodecen-11-olide, 5- and 6-tridecen-12-olide, 5- to 9-tetradecen-13-olide, as well as 5- and 6-pentadec-14-olide (see Supporting Information File 1 for some spectra). This set of mass spectra allowed the identification of macrolides in other frog species.

We also found macrolide **1** in the gular glands of other hyperoliids including *H. concolor*, *H. adametzi*, and *Afrixalus dorsalis*. It occurs also outside this frog family. It is a major constituent of the femoral gland of the mantellid frog *Spinomantis aglavei,* again in (*S*)*-*configuration. Minor amounts of **1** were also present in the glands of an undescribed species of *Guibemantis* similar to *G. bicalcaratus* as well as in *Gephyromantis ceratophrys*, accompanied by the isomer 8-tetradecen-13-olide in the latter species.

## Conclusion

RCM using the (*Z*)-selective Grubbs catalyst **12** is a convenient strategy to prepare unsaturated macrocyclic lactones in a short sequence. Although isomerization is usually regarded as a disadvantage of RCM, it can be used to allow fast access to mass spectra of several isomers, helpful for the structure elucidation of natural compounds, e.g., in chemical ecology or fragrance research. The mass spectral fragmentation of macrolides differs markedly from that of open-chain esters, because initial bond cleavage often does not lead to the release of an uncharged radical, but to the formation of a distonic cationic radical prone to further fragmentation. The analysis of the fragmentation led to a rationale for the determination of the bound bond position in unsaturated macrolides. The identification of **1** and **5** in the gular gland of *H. cinnamomeoventris* and other species underlines the importance of macrolides for the chemical ecology of hyperoliid and mantellid frogs. On the contrary, other compounds such as the terpenes commonly found in hyperoliids remain largely unknown. Their identification and synthesis are a priority and would constitute a major step towards a biological evaluation of the gland secretion and its compounds to understand their real function in the behavior of the frogs. Although no experimental evidence has been obtained so far, the close association of the innervation of the gland with the mating period, its location and use during calling, and its male-specific occurrence strongly hint to a function of **1** and **5** as signaling compounds and a role in the chemical ecology of this species.

## Supporting Information

File 1 Experimental procedures, mass spectra of macrolides, alternative fragmentation pathway, enantiomer separation by GC–MS, ^1^H and ^13^C NMR spectra.
