# Supplementary material for: Identification, synthesis and mass spectrometry of a macrolide from the African reed frog Hyperolius cinnamomeoventris
Source: Beilstein J Org Chem. 2016 Dec 13;12:2731–8. doi: 10.3762/bjoc.12.269 (PMC5238529; doi:10.3762/bjoc.12.269)
Supplement: File 1 — Experimental procedures, mass spectra of macrolides, alternative fragmentation pathway, enantiomer separation by GC–MS, 1H and 13C NMR spectra. [file Beilstein_J_Org_Chem-12-2731-s001.pdf]

Supporting Information

for

Identification, synthesis and mass spectrometry of a macrolide from the African reed frog *Hyperolius cinnamomeoventris*

Markus Menke[1,§], Pardha Saradhi Peram[1,§], Iris Starnberger[2],Walter Hödl[2], Gregory F.M. Jongsma[3], David C. Blackburn[3], Mark-Oliver Rödel[4], Miguel Vences[5] and Stefan Schulz[1,*]

Address: [1]Technische Universität Braunschweig, Institute of Organic Chemistry, Hagenring 30, 38106 Braunschweig, Germany, [2]Department for Integrative Zoology, Althanstraße 14, 1090 Vienna, Austria, [3]Florida Museum of Natural History, University of Florida, Gainesville, Florida 32611, United States of America, [4]Museum für Naturkunde, Leibniz Institute for Evolution and Biodiversity Science, Invalidenstr. 43, 10115 Berlin, Germany and [5]Technische Universität Braunschweig, Institute of Zoology, 38106 Braunschweig, Germany

Email: Stefan Schulz - stefan.schulz@tu-bs.de

* Corresponding author

[§]Both authors share first authorship.

Experimental procedures, mass spectra of macrolides, alternative fragmentation pathway, enantiomer separation by GC–MS, ^{1}H and ^{13}C NMR spectra

General experimental methods

Chemicals have been acquired from Acros Organics, Sigma-Aldrich, Alfa Aesar and Merck. Reactions with water and/or air unstable reagents were performed in flame dried flasks under a nitrogen atmosphere. Conventionally dried solvents were distilled before use.

^{1}H-NMR- and ^{13}C-NMR-spectra were acquired on the following instruments (Bruker): DPX-200 (200 MHz for ^{1}H and 50 MHz for ^{13}C), AV II-300 (300 MHz for ^{1}H and 75 MHz for ^{13}C), DRX-400 (400 MHz for ^{1}H and 100 MHz for ^{13}C), AV III-400 (400 MHz for ^{1}H und 100 MHz for ^{13}C) und AV II-600 (600 MHz for ^{1}H and 150 MHz for ^{13}C). Tetramethylsilane was used as an internal standard (TMS, δ = 0 ppm). Multiplicities of the protons are described as singulets (s), doublets (d), triplets (t), quartets (q), quintets (quint), sextets (sext), septets (sept), or multiplets (m). The connectivities of the carbon atoms are described as primary (CH_3), secondary (CH_2), tertiary (CH) or quarternary (C_q).

GC–MS analyses of synthetic products were performed on a GC HP6890/MSD HP5973 combination (Hewlett Packard) and natural samples on a GC 7890A/MSD 5975C from Agilent. Mass spectrometry was performed in electron ionization mode (EI) with 70 eV. Fused-silica capillary columns BPX-5 (SGE Inc., 25 m, 0.22 mm i.d. 0.25 µm film thickness) and HP-5MS (Agilent Technologies, 30 m, 0.25 mm i.d. 0.25 µm film thickness) were used with helium as the carrier gas.

High resolution mass spectrometry data were obtained with a GC 6890 gas chromatograph (Agilent Technologies) equipped with a Phenomenex ZB5-MS column (30 m × 0.25 mm i.d. × 0.25 µm) coupled with a time-of-flight mass spectrometer JMS-T100GC, GCAccuTOF (JEOL, Japan) in EI mode (70 eV). JEOL MassCenter Workstation Software was used. The instrument was calibrated with PFK to reach a resolution of 5000 (fwhm) at m/z = 292.9824.

Chiral gas chromatography was performed with a Hydrodex-6-TBDMS (25 m × 0.25 mm i.d., Macherey-Nagel).

IR-spectra were acquired on Tensor 27 (Bruker) with diamond-ATR-technique. The peaks are listed with wave numbers in cm^{-1}. Intensities are described with s (strong), m (medium), w (weak) and br (broad).

Purification of synthetic compounds with column chromatography has been performed with silica from Fluka (silica gel 60, particle size 0.040–0.063 mm, Mesh 230–440 ASTM) using ethyl acetate, pentane, and diethyl ether as solvent.

Thin layer chromatography has been performed using silica coated plate Polygram SIL G/UV254 from Macherey & Nagel using molybdate phosphoric acid for detection.

Optical rotation was measured on a Dr. Kernchen Propol Digital Automatic polarimeter with 1 cm cuvettes at a wavelength of 589 nm.

Natural samples

Specimens of hyperoliid and mantellid frogs, mostly calling males, were collected in the field during the reproductive season. They were euthanised using anesthesia and subsequent overdose with MS222 (ethyl 3-aminobenzoate methanesulfonate). Individuals from Rwanda were treated similarly with benzocaine. Subsequently, gland tissue from the gular region (gular gland) or ventral femoral skin (femoral glands) was excised and preserved in dichlormethane. Voucher specimens were fixed in 95% ethanol and preserved in 70% ethanol, and deposited in the collections of the Zoologische Staatssammlung München, Germany, the Université d'Antananarivo, Département de Biologie Animale, Madagascar, and the California Academy of Sciences (San Francisco, California, USA).

General procedure for ring-closing metathesis attempts using Grubbs–Hoveyda II catalyst and the (*Z*)-selective Grubbs catalyst

The diene ester (50 mg, 0.198 mmol, 1 equiv) in 67 mL toluene, C_6F_6 (368 mg, 1.98 mmol, 10 equiv), *p*-benzoquinone (2 mg, 0.0185 mmol, 0.15 equiv) and the catalyst (12 mg for **11**, 12.5 mg for **12**, 0.0198 mmol, 0.1 equiv) were heated to 80 °C for 3 h. The toluene was removed in vacuo and column chromatography (pentane/diethyl ether, 30:1) yielded the pure product [S1-S4].

(*R*)-1,2-Epoxyhex-5-ene (*R*-6)

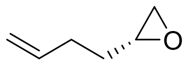

(*R*)-**6**

Initially, toluene was removed from a solution of (*R*,*R*)-(−)-*N,N′*-bis(3,5-di-*tert*-butylsalicylidene)-1,2-cyclohexanediaminocobalt(III)·OAc catalyst (**10**, 0.5 mol, 0.5 equiv, 0.55 mL of a 0.0916 M solution in toluene). The catalyst was added to 1,2-epoxyhex-5-ene (**6**, 976 mg, 9.94 mmol, 1 equiv) in 0.3 mL Et_2O. The solution was cooled to 0 °C, 10 µL water (100 mg, 5.5 mmol, 0.55 equiv) was added, and the solution was stirred for 1 h at 0°C. After stirring overnight at room temperature the catalyst was removed by filtration over a short column of silica (pentane/diethyl ether, 10:1). The crude product was distilled via bulb-to-bulb-transfer into a −100 °C cooled flask in vacuo to get 810 mg of a mixture of the (*R*)-**6** and Et_2O ready to use in the next step. Further removal of Et_2O would have led to significant loss of product. The yield was evaluated by NMR (672 mg, 6.86 mmol, 69%) [S5,S6].

TLC — pentane/diethyl ether (10:1):
R_F — 0.52

^{1}H-NMR — (300 MHz, $CDCl_3$):
δ [ppm] — 5.84 (1 H, ddt, ^3J=16.9 Hz, ^3J=10.2 Hz, ^3J=6.7 Hz), 5.06 (1H, ddt, ^2J= 3.2 Hz, ^3J= 17.3 Hz, ^4J= 1.7 Hz) 5.02 (1 H, ddt, ^2J= 2.1 Hz, ^3J= 10.2 Hz, ^4J= 1.3 Hz) , 2.98-2.88 (1 H, m), 2.77 (1 H, dd, ^2J= 5.0 Hz, ^3J= 3.0 Hz), 2.50 (1 H, dd, ^2J= 5.0 Hz, ^3J= 3.0 Hz), 2.28-2.18 (2 H, m) 1.70-1.58 (2 H, m).

^{13}C-NMR — (75 MHz, $CDCl_3$):
δ [ppm] — 137.6 (1 C, CH), 115.1 (1 C, CH_2), 51.8 (1 C, CH), 47.1 (1 C, CH_2), 31.8 (1 C, CH_2), 30.1 (1 C, CH_2).

IR (Diamant ATR):
$\tilde{\nu}$ [cm^{-1}] — 3078 (w), 3048 (w), 2981 (w), 2925 (w), 2848 (w), 1641 (m), 1484 (w), 1444 (w), 1141 (w), 1262 (w), 1132 (w), 997 (m), 912 (s), 837 (s), 741 (w) 636 (w).

GC/MS — (EI = 70 eV):
m/z (%) — 97 (9, [M$^+$-1]), 83 (23), 79 (23), 68 (45), 67 (100), 55 (54), 54 (55), 41 (58), 39 (81).

Optical rotation product (R):
$[\alpha]_D^{22.1} = +14.0$, (c= 1.0 in Et_2O) [Lit.: $[\alpha]_D^{25} = +9.36$, c = neat [S6]]

Optical rotation starting material:
$[\alpha]_D^{21.7} = +5.7$, (c= 1.0 in Et_2O)

(*R*)-Hex-5-en-2-ol ((*R*)-7)

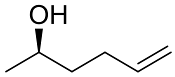

(*R*)-**7**

(*R*)-1,2-Epoxyhex-5-ene ((*R*)-**6**, 417 mg, 4.26 mmol, 1 equiv) was added slowly to an ice cold solution of $LiAlH_4$ (206 mg, 5.14 mmol, 1.2 equiv) in 20 mL Et_2O. The mixture was stirred for 1 h at 0 °C. After stirring overnight, sat. NH_4Cl-solution was added. The aqueous phase was extracted three times with Et_2O. The organic phase was washed with brine, dried with $MgSO_4$ and most Et_2O was removed in vacuo. The product still contained Et_2O which removal would have led to significant loss of the product. The yield was estimated by NMR (384 mg, 3.84 mmol, 90%) [S7].

^{1}H-NMR (400 MHz, $CDCl_3$):
δ [ppm] 5.84 (1 H, ddt, ^{3}J= 16.9 Hz, ^{3}J= 10.2 Hz, ^{3}J = 6.6 Hz), 5.05 (1 H, ddt, ^{2}J= 3.6 Hz, ^{3}J= 17.1 Hz, ^{4}J= 1.7 Hz), 4.96 (1 H, ddt, ^{2}J= 2.1 Hz, ^{3}J= 10.1 Hz, ^{4}J= 1.3 Hz), 3.82 (1 H, dq, ^{3}J= 12.4 Hz, ^{3}J= 6.2 Hz), 2.21-2.10 (2 H, m), 1.79 (1 H, br. s.), 1.61-1.49 (2 H, m), 1.20 (3 H, d, ^{3}J=6.3 Hz).

^{13}C-NMR (100 MHz, $CDCl_3$):
δ [ppm] 138.5 (1 C, CH), 114.6 (1 C, CH_2), 67.5 (1 C, CH), 38.2 (1 C, CH_2), 30.1 (1 C, CH_2), 23.4 (1 C, CH_3).

IR (Diamant ATR):
$\tilde{\nu}$ [cm^{-1}] 3345 (br. s.), 3079 (w), 2970 (m), 2929 (m), 1641 (m), 1453 (m), 1374 (w), 1308 (w), 1122 (m), 994 (m), 908 (s), 846 (m), 636 (m).

GC/MS (EI = 70 eV):
m/z (%) 99 (0, [M^+-1]), 82 (45, [M^+-18]), 71 (12), 67 (100), 58 (23), 56 (27), 45 (86), 41 (41), 39 (26).

Optical rotation (R):
$[\alpha]_D^{21.7} = -15.9$, (c= 0.48 in Et_2O) [Lit.: $[\alpha]_D^{25} = -12.1$, c = 4.6 in $CHCl_3$ [S8]]

Synthesis of (*R*)-hex-5-en-2-yl dec-9-enoate ((*R*)-9)

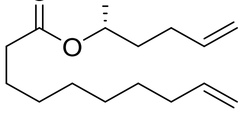

(*R*)-**9**

A solution of (*R*)-hex-5-en-2-ol ((*R*)-**7,** 264 mg, 2.64 mmol, 1 equiv), 9-decenoic acid (493 mg, 2.90 mmol, 1.1 equiv) and 4-dimethylaminopyridine (DMAP, 32 mg, 0.264 mmol, 0.1 equiv) in 50 mL CH_2Cl_2 is cooled to 0 °C. *N*-(3-Dimethylaminopropyl)-*N*'-ethylcarbodiimide hydrochloride (EDC, 505 mg, 2.64 mmol, 1 eq.) was added and the mixture was stirred for 1 h at 0 °C. After stirring for 72 h at room temperature a sat. $NaHCO_3$-solution was added. The aqueous phase was extracted three times with CH_2Cl_2, the organic phase was washed with brine, dried with $MgSO_4$ and the solvent was removed in vacuo. After column chromatography (pentane/ether, 30:1) the product was isolated as a colorless liquid (463 mg, 1.64 mmol, 62%) [S9].

TLC pentane/diethyl ether (30:1):
R_F 0.21

^{1}H-NMR (300 MHz, $CDCl_3$):
δ [ppm] 5.88-5.73 (2 H, m), 5.05-4.86 (5 H, m), 2.27 (2 H, t, ^3J = 7.5 Hz), 2.15-1.99 (4 H, m), 1.77-1.49 (4 H, m), 1.43-1.24 (8 H, m), 1.21 (3 H, d, ^3J= 6.3 Hz)

^{13}C-NMR (75 MHz, $CDCl_3$)
δ [ppm] 173.4 (1 C, C_q), 139.1 (1 C, CH), 137.8 (1 C, CH), 114.9 (1 C, CH_2), 114.2 (1 C, CH_2), 70.1 (1 C, CH), 35.1 (1 C, CH_2), 34.7 (1 C, CH_2), 33.7 (1 C, CH_2), 29.7 (1 C, CH_2), 29.1 (2 C, CH_2), 28.9 (1 C, CH_2), 28.8 (1 C, CH_2), 25.0 (1 C, CH_2), 20.0 (1 C, CH_3).

IR (diamond-ATR):
$\tilde{\nu}$ [cm^{-1}] 3078 (w), 2977 (w), 2929 (m), 2857 (w), 1731 (s), 1641 (w), 1458 (w), 1377 (w), 1246 (m), 1175 (s), 1126 (m), 993 (m), 909 (s), 725 (w), 635 (w).

GC/MS (EI = 70 eV)
m/z (%) 252 (0, [M^+]), 152 (11), 135 (38), 123 (7), 110 (15), 96 (11), 83 (60), 82 (90), 69 (42), 67 (92), 55 (100), 41 (55).

Optical rotation (*R*):
$[\alpha]_D^{22.9} = -6.7$, (c= 0.86 in CH_2Cl_2)

Synthesis of (9*Z*,13*R*)-9-tetradecene-13-olide ((*R*)-2)

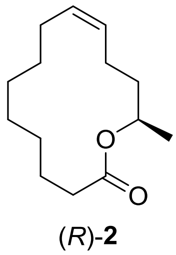

(*R*)-**2**

via Grubbs–Hoveyda-II-catalyst (11)
According to the general procedure, a mixture of several isomers of the desired macrolide was obtained in 27% yield (12 mg, 0.054 mmol) using [1,3-bis-(2,4,6-trimethylphenyl)-2-imidazolidinylidene]dichloro(*o*-isopropoxyphenylmethylene)ruthenium (**11**, Grubbs–Hoveyda II catalyst).

via (*Z*)-selective Grubbs-catalyst (12)

According to the general procedure, (9*Z*,13*R*)-9-tetradecene-13-olide was obtained in 38% yield (17 mg, 0.076 mmol, 74% brsm) with the catalyst [2-(1-methylethoxy-*O*)phenylmethyl-*C*](nitrato-*O,O'*){*rel*-(2*R*,5*R*,7*S*)-tricyclo[3.3.1.13,7]decane-2,1-diyl[3-(2,4,6-trimethylphenyl)-1-imidazolidinyl-2-ylidene]}ruthenium (**12**, (*Z*)-selective Grubbs-catalyst).

TLC pentane/diethyl ether (30:1)
R_F 0.14

^{1}H-NMR (300 MHz, $CDCl_3$)
δ [ppm] 5.50-5.32 (2 H, m), 5.11-5.02 (1 H, m), 2.49-1.18 (21 H, m).

^{13}C-NMR (75 MHz, $CDCl_3$)
δ [ppm] 173.6 (1 C, C_q), 130.3 (1 C, CH), 129.1 (1 C, CH), 69.8 (1 C, CH), 36.0 (1 C, CH_2), 33.6 (1 C, CH_2), 26.9 (1 C, CH_2), 26.6 (1 C, CH_2), 26.1 (1 C, CH_2), 25.2 (1 C, CH_2), 24.9 (1 C, CH_2), 24.6 (1 C, CH_2), 22.1 (1 C, CH_2), 19.5 (1 C, CH_3).

IR (diamond-ATR)
$\tilde{\nu}$ [cm^{-1}] 3004 (w), 2930 (s), 2858 (m), 1732 (s), 1460 (w), 1371 (w), 1215 (w), 1178 (w), 1131 (w), 1106 (w), 720 (w).

GC/MS (EI = 70 eV)
m/z (%) 224 (40, [M^+]), 182 (17), 164 (11), 150 (9), 135 (13), 122 (19), 109 (27), 95 (74), 82 (77), 81 (81), 68 (68), 67 (100).

Optical rotation (*R*)
$[\alpha]_D^{22.9} = -40.1$, c = 1.05 (CH_2Cl_2)

Synthesis of dec-9-en-2-ol (15)

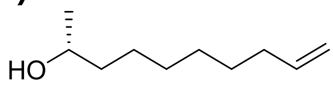

15

7-Bromo-1-heptene (**14**, 1 g, 5.65 mmol, 1.3 equiv) was added to a suspension of I_2-activated Mg (137 mg, 5.65 mmol, 1.3 equiv) in 9 mL THF. After heating to reflux for 1 h, the solution was stirred for an additional 4.5 h at room temperature. The Grignard-solution was then added to a mixture of CuBr·SMe_2 (89 mg, 0.434 mmol, 0.1 equiv) in 6 mL THF at −78 °C. After stirring for 20 min (*R*)- or (*S*)-propylene oxide (**13**, 252 mg, 4.34 mmol, 1.0 equiv) was added and the mixture was stirred overnight at room temperature. Sat. NH_4Cl solution was added, the aqueous phase was extracted three times with EtOAc, and the organic phase was dried with $MgSO_4$. After column chromatography (pentane/EtOAc, 4:1) the product was obtained as a colorless liquid [S10].
Yield for the (*R*)-enantiomer: 615 mg, 3.94 mmol, 91%, Yield for the (*S*)-enantiomer: 564 mg, 3.61 mmol, 83%).

TLC pentane/EtOAc (4:1)
0.41

^{1}H-NMR (400 MHz, $CDCl_3$)
δ [ppm] 5.81 (1 H, ddt, ^{3}J= 16.9 Hz, ^{3}J = 10.2 Hz, ^{3}J= 6.7 Hz), 4.99 (1 H, ddt, ^{2}J= 2.1 Hz, ^{3}J= 17.1 Hz, ^{4}J= 1.7 Hz) 4.93 (1 H, ddt, ^{2}J= 2.1 Hz, ^{3}J= 10.1 Hz, ^{4}J= 1.3 Hz) 3.84-3.74 (1 H, m), 2.07-2.00 (2 H, m) , 1.54-1.26 (11 H, m), 1.18 (3 H, d, ^{3}J = 6.3 Hz).

^{13}C-NMR (100 MHz, $CDCl_3$)
δ [ppm] 139.1 (1 C, CH), 114.2 (1 C, CH_2), 68.2 (1 C, CH), 39.3 (1 C, CH_2), 33.8 (1 C, CH_2), 29.5 (1 C, CH_2), 29.1 (1 C, CH_2), 28.8 (1 C, CH_2), 25.7 (1 C, CH_2), 23.5 (1 C, CH_3).

IR (diamond-ATR)
$\tilde{\nu}$ [cm^{-1}] 3343 (br. s.), 3077 (w), 2968 (m), 2926 (s), 2855 (m), 1641 (w), 1461 (m), 1373 (w), 1307 (w), 1122 (m), 993 (m), 908 (s), 840 (w), 635 (w).

GC/MS (EI = 70 eV)
m/z (%) 156 (1 [M^+]), 138 (4, [M^+-18]), 123 (9), 110 (26), 96 (48), 95 (32), 81 (80), 67 (68), 55 (80), 45 (100), 41 (69).

Optical rotation (*S*):
$[\alpha]_D^{21.7} = +7.9$, c = 1.10 (CH_2Cl_2)

Optical rotation (*R*):
$[\alpha]_D^{21.7} = -4.7$, c = 0.98 (CH_2Cl_2), [Lit.: $[\alpha]_D^{25} = -19.8$, c = 1.87 (CH_2Cl_2) [S10]]

Synthesis of dec-9-en-2-yl hex-5-enoate (17)

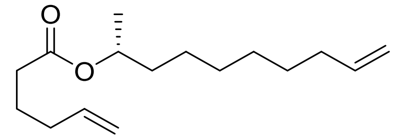

17

A solution of dec-9-en-2-ol (**15**, 400 mg, 2.56 mmol, 1 equiv), 5-hexenoic acid (**16**, 322 mg, 2.82 mmol, 1.1 equiv) and DMAP (31 mg, 0.256 mmol, 0.1 equiv) in 50 mL CH_2Cl_2 was cooled to 0 °C. *N*-(3-Dimethylaminopropyl)-*N*'-ethylcarbodiimide hydrochloride (EDC, 492 mg, 2.56 mmol, 1 equiv) was added and the mixture was stirred for 1 h at 0 °C. After stirring for 72 h at room temperature, sat. $NaHCO_3$ solution was added. The aqueous phase was extracted three times with CH_2Cl_2, the organic phase was washed with brine, dried with $MgSO_4$ and the solvent was removed in vacuo. After column chromatography (pentane/diethyl ether, 30:1) the product was isolated as a colorless liquid [S9].
Yield for the (*R*)-enantiomer: 389 mg, 1.54 mmol, 60%, Yield for the (*S*)-enantiomer: 380 mg, 1.51 mmol, 59%).

TLC	pentane/diethyl ether (30:1)
R_F	0.23

^{1}H-NMR	(400 MHz, $CDCl_3$):
δ [ppm]	5.87-5.72 (2 H, m), 5.07-4.84 (5 H, m), 2.29 (2 H, t, ^3J= 7.4 Hz), 2.13-1.99 (4 H, m), 1.72 (2 H, qui, ^3J = 7.4 Hz), 1.63-1.23 (10 H, m), 1.19 (3 H, d, ^3J= 6.3 Hz).

^{13}C-NMR	(100 MHz, $CDCl_3$)
δ [ppm]	173.2 (1 C, C_q), 139.1 (1 C, CH), 137.8 (1 C, CH), 115.3 (1 C, CH_2), 114.2 (1 C, CH_2), 70.8 (1 C, CH), 35.9 (1 C, CH_2), 34.0 (1 C, CH_2), 33.7 (1 C, CH_2), 33.1 (1 C, CH_2), 29.3 (1 C, CH_2), 29.0 (1 C, CH_2), 28.8 (1 C, CH_2), 25.3 (1 C, CH_2), 24.2 (1 C, CH_2), 20.0 (1 C, CH_3).

IR	(diamond-ATR)
$\tilde{\nu}$ [cm^{-1}]	3078 (w), 2977 (m), 2929 (s), 2857 (m), 1731 (s), 1641 (w), 1458 (w), 1377 (w), 1246 (w), 1224 (w), 1175 (m), 1126 (m), 993 (m), 909 (s), 635 (w).

GC/MS	(EI = 70 eV)
m/z (%)	252 (<1 %, [M^+]), 237 (1), 192 (1), 165 (1), 138 (15), 123 (3), 114 (71), 110 (24), 97 (100), 96 (64), 81 (50), 69 (85), 68 (71), 55 (97), 41 (81).

Optical rotation (*S*)
$[\alpha]_D^{21.8} = +7.0$, c = 0.87 (CH_2Cl_2)

Optical rotation (*R*)
$[\alpha]_D^{21.8} = -3.3$, c = 0.90 (CH_2Cl_2)

Synthesis of (*Z*)-5-tetradecen-13-olide (1)

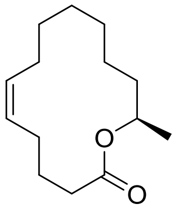

1

According to General Procedure macrolide **1** was obtained after heating to reflux for 24 h in toluene with the (*Z*)-selective Grubbs-catalyst **12**.
Yield for (*R*)-enantiomer: 14 mg, 0.062 mmol, 31%, Yield for (*S*)-enantiomer: 16 mg, 0.071 mmol, 36%).

TLC pentane/diethyl ether (30:1)
R_F 0.19

^{1}H-NMR (300 MHz, $CDCl_3$)
δ [ppm] 5.45-5.26 (2 H, m), 5.05-4.90 (1 H, m), 2.49 - 2.13 (4 H, m), 1.98-1.10 (14 H, m), 1.23 (3 H, d, ^3J= 6.3 Hz).

^{13}C-NMR (75 MHz, $CDCl_3$)
δ [ppm] 173.4 (1 C, C_q), 130.8 (1 C, CH), 128.8 (1 C, CH), 69.2 (1 C, CH), 34.6 (1 C, CH_2), 33.8 (1 C, CH_2), 26.9 (1 C, CH_2), 26.6 (1 C, CH_2), 26.2 (1 C, CH_2), 25.2 (1 C, CH_2), 24.9 (2 C, CH_2), 23.2 (1 C, CH_2), 20.7 (1 C, CH_3).

IR (diamond-ATR)
$\tilde{\nu}$ [cm^{-1}] 3000 (w), 2928 (m), 2858 (m), 1728 (s), 1459 (w), 1374 (w), 1291 (m), 1244 (m), 1206 (m), 1156 (w), 1130 (m), 1041 (m), 1016 (w), 906 (w), 876 (w), 806 (w), 705 (w).

GC/MS (EI = 70 eV)
m/z (%) 224 (30, [M^+]), 209 (2, [M^+-15]), 206 (4, [M^+-18]), 195 (2), 181 (8), 164 (17), 150 (13), 135 (16), 126 (42), 124 (24), 110 (30), 95 (47), 82 (58), 81 (100), 67 (91), 55 (73), 41 (87).

Optical rotation (*S*)
$[\alpha]_D^{22.4} = +42.3$, c = 0.95 (CH_2Cl_2) Lit.: $[\alpha]_D^{23} = +48.1$, c = 1.35 ($CHCl_3$) [S11]]

Optical rotation (*R*)
$[\alpha]_D^{22.4} = -31.2$, c = 0.88 (CH_2Cl_2)

Enantiomeric excess (chiral GC)
Hydrodex β-6TBDMS phase: Temperature program: isothermal for 60 min at 110 °C, then with 2 °C/min to 160 °C, followed by a sharp ramp with 25 °C/min to 220 °C.
(*S*) *t*= 83.65 min 98%
(*R*) *t*= 84.40 min 99%

Alternative mass spectrometric fragmentation pathway to characteristic ions of unsaturated macrolides

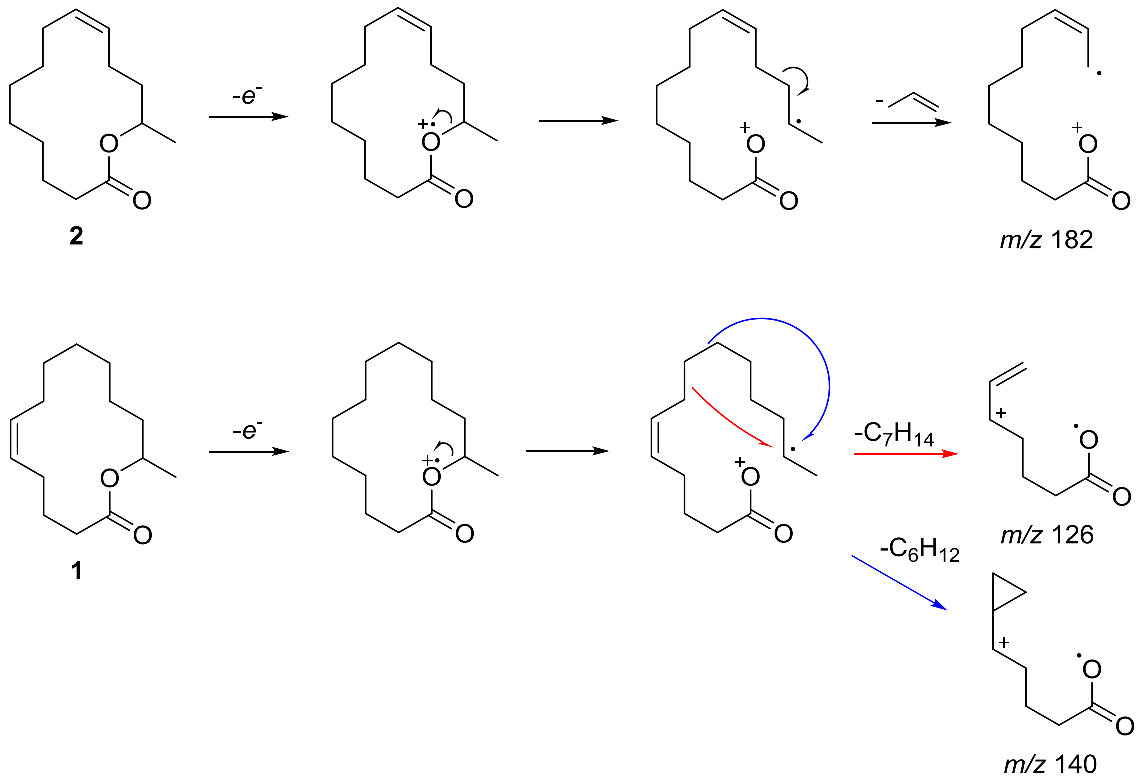

Figure S1. Alternative mass spectrometric fragmentation of macrolides **1** and **2** leading to diagnostic ions of the ion series $C_nH_{2n-4}O_2$ ($C_{n+4}H_{2n+4}O_2$ in the figure), indicating the position of the double bond in the ring. The other pathway is shown in Figure 5 of the main article. The proposals differ in the initial position of the radical and the positive charge.

Mass spectra of other macrolide isomers

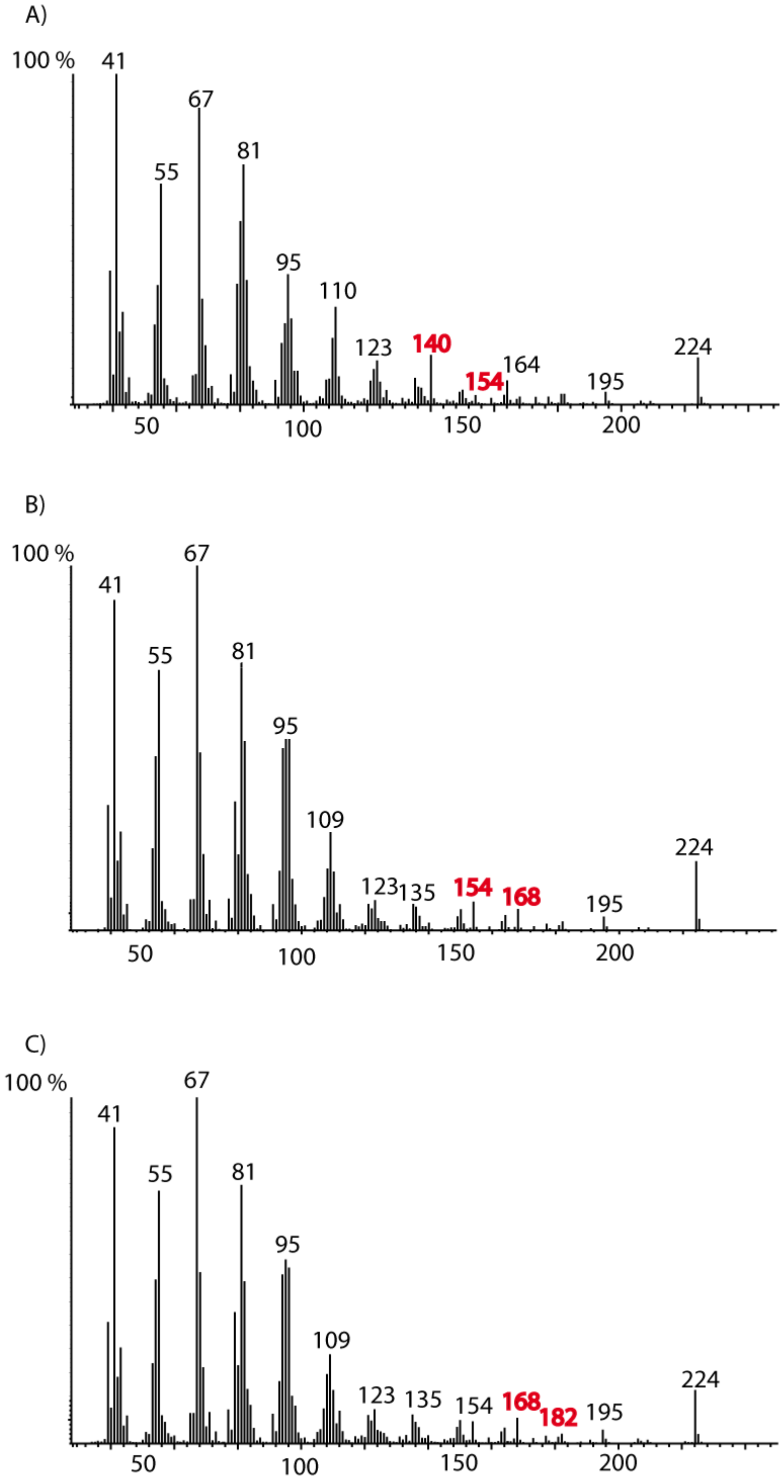

Figure S2: Mass spectra of A) (*Z*)-6-tetradecen-13-olide, B) (*Z*)-7-tetradecen-13-olide, C) (*Z*)-8-tetradecen-13-olide

GC on chiral phase

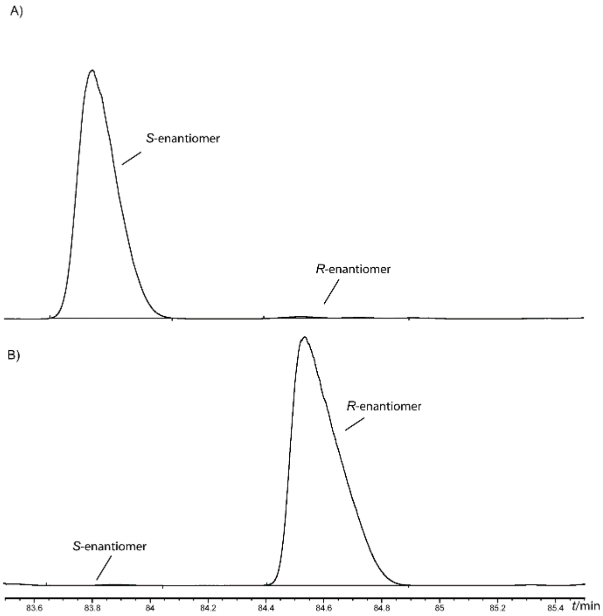

Figure S3: Gas chromatographic separation of (Z)-5-tetradecen-13-olide (**1**) on a chiral Hydrodex β-6TBDMS phase. A) (*S*) ee 98%, B) (*R*) ee 99%. Temperature program: isothermal for 60 min at 110 °C, then with 2 °C/min to 160 °C, followed by a sharp ramp with 25 °C/min to 220 °C.

^{1}H NMR and ^{13}C NMR spectra of compounds 1,2,6,7,9,15 and 17

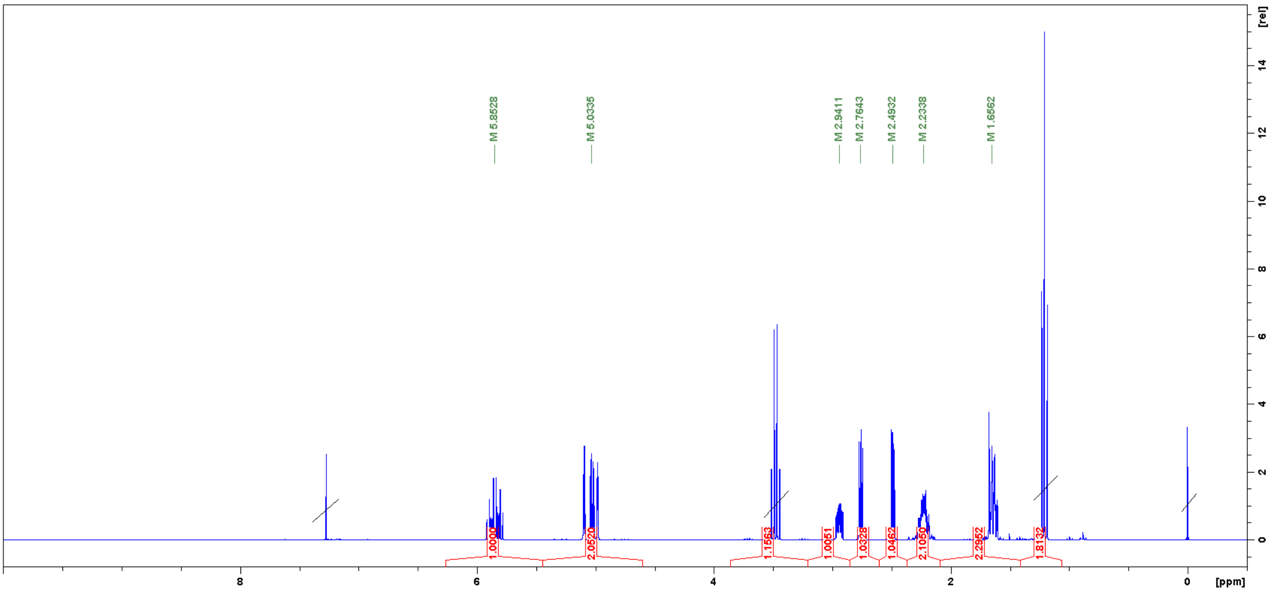

Figure S4: ^{1}H NMR spectrum of 1,2-epoxyhex-5-ene (**6**).

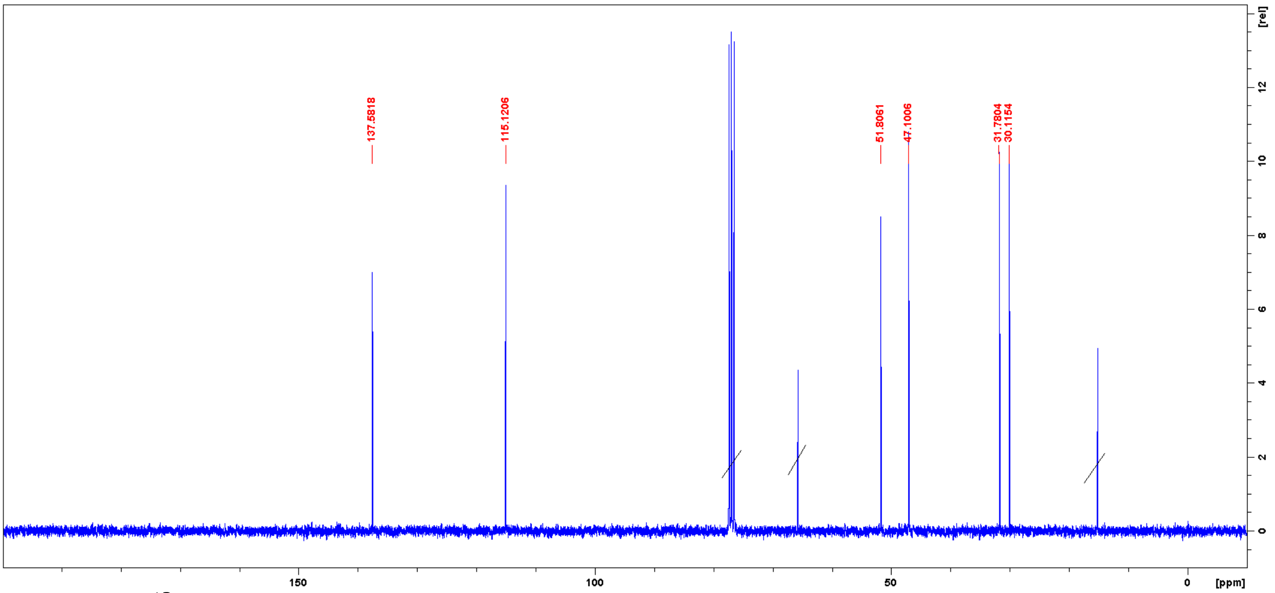

Figure S5: ^{13}C NMR spectrum of 1,2-epoxyhex-5-ene (**6**).

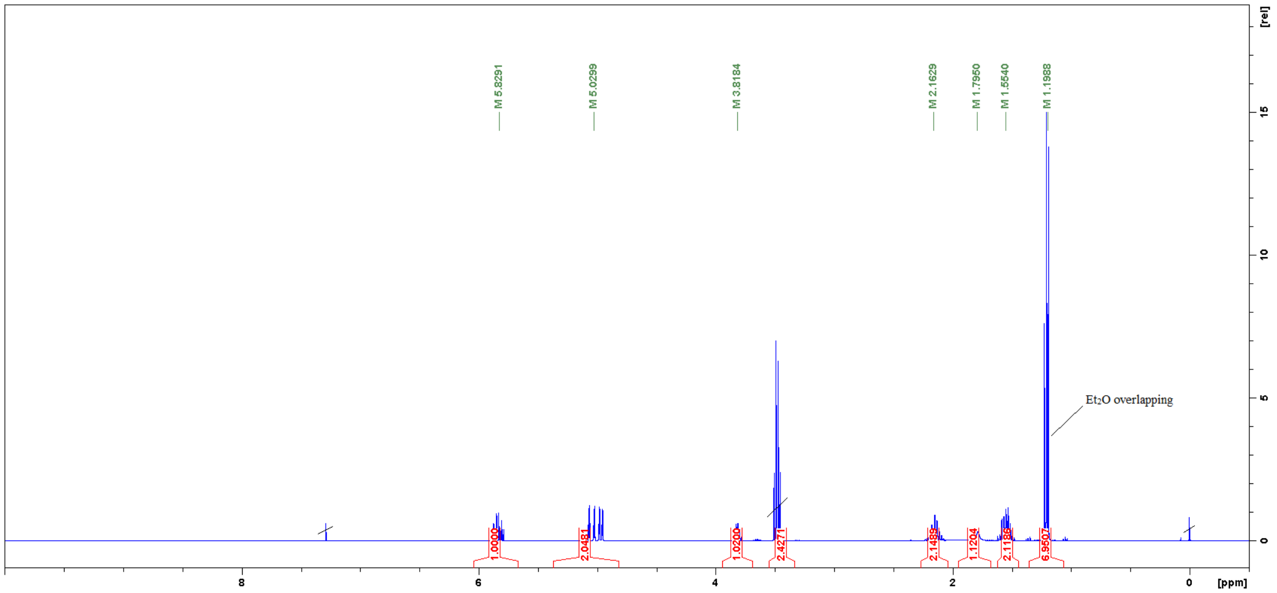

Figure S6: ^{1}H NMR spectrum of hex-5-en-2-ol (**7**).

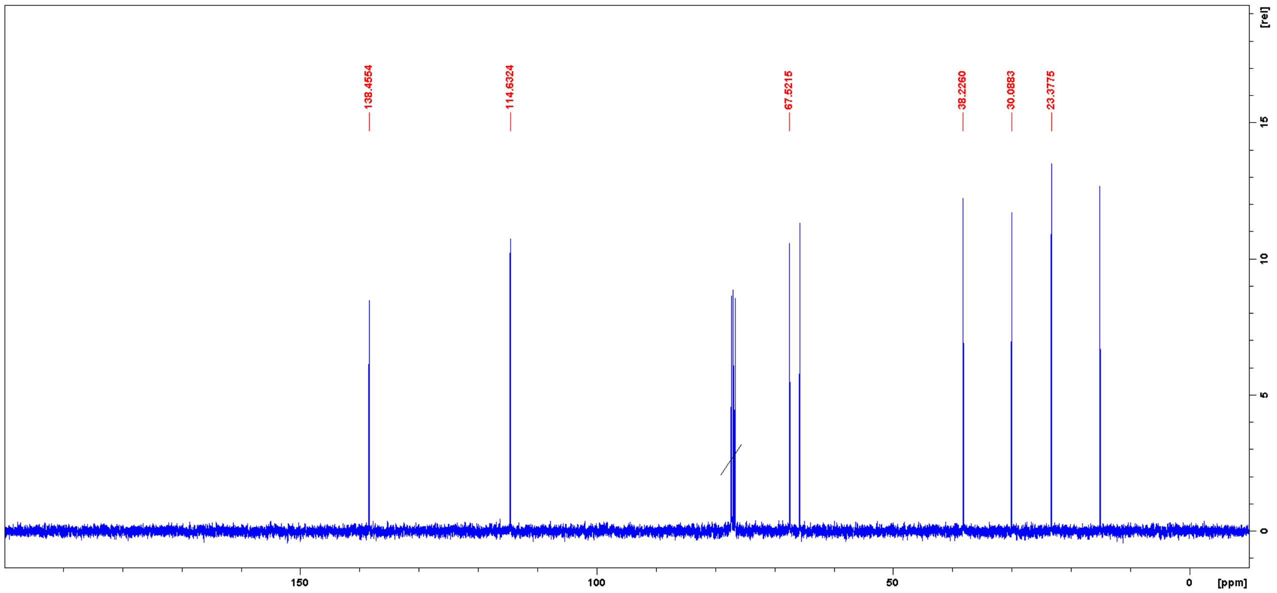

Figure S7: ^{13}C NMR spectrum of hex-5-en-2-ol (**7**).

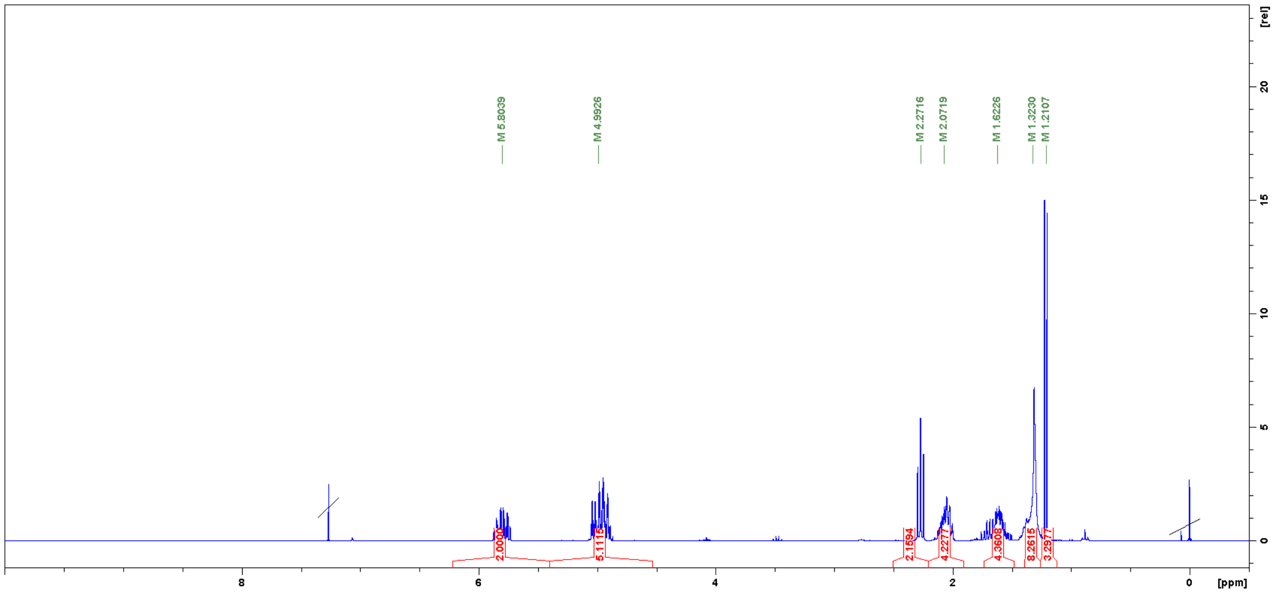

Figure S8: ^{1}H NMR spectrum of hex-5-en-2-yl dec-9-enoate (**9**).

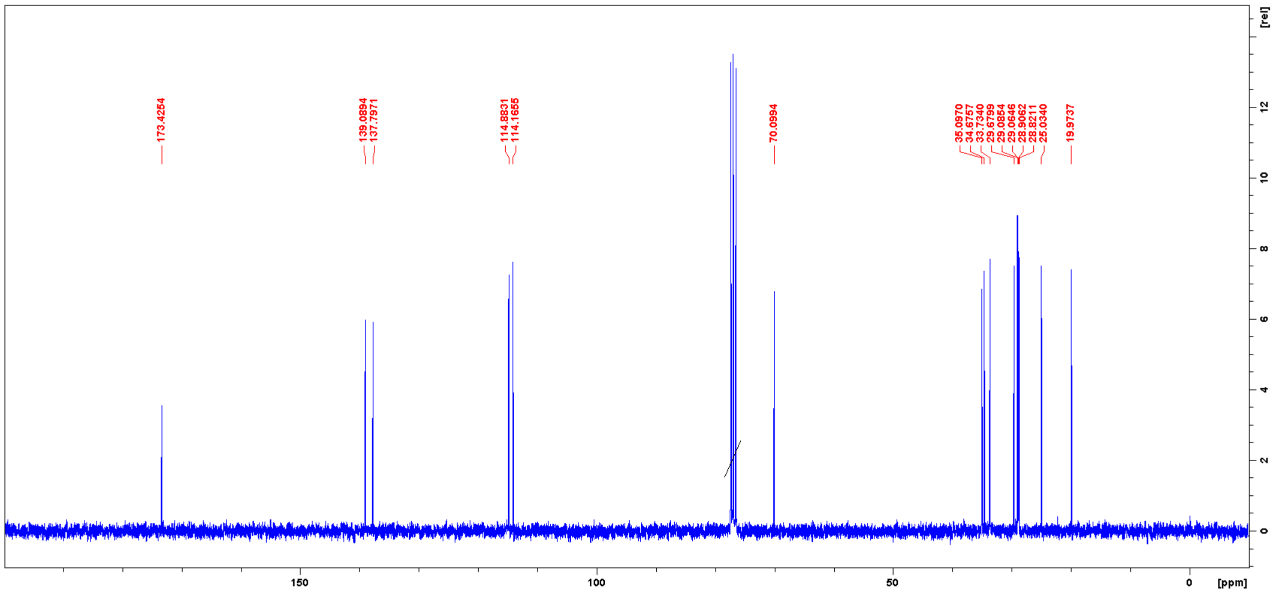

Figure S9: ^{13}C NMR spectrum of hex-5-en-2-yl dec-9-enoate (**9**).

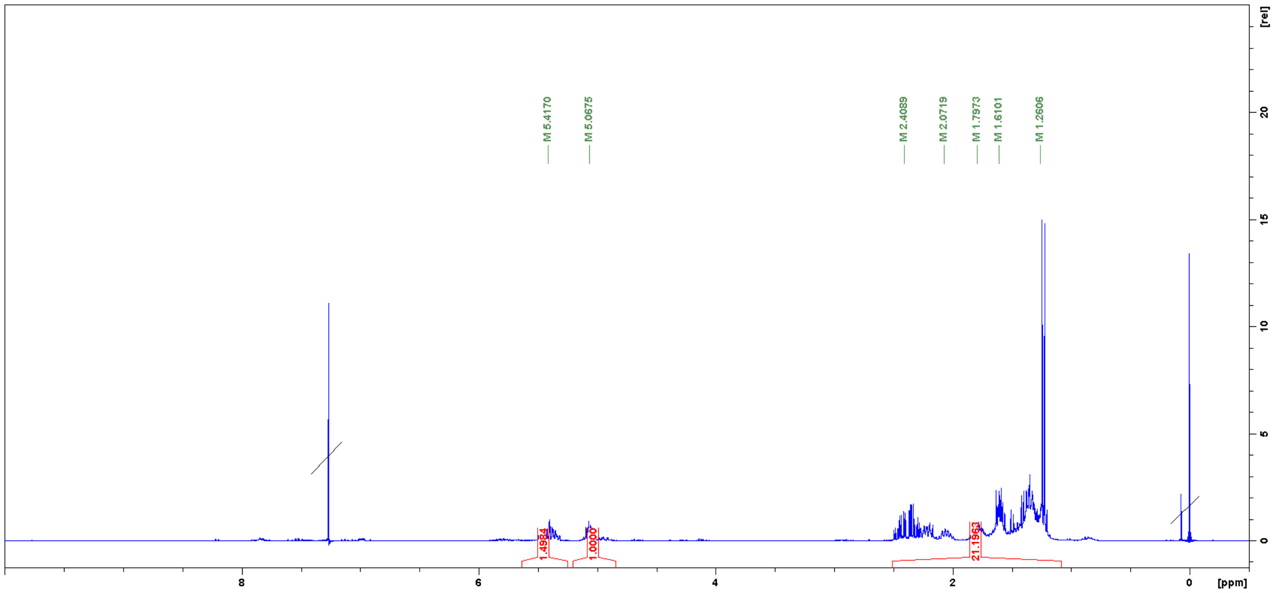

Figure S10: ^{1}H NMR spectrum of (*Z*)-9-tetradecen-13-olide (**2**).

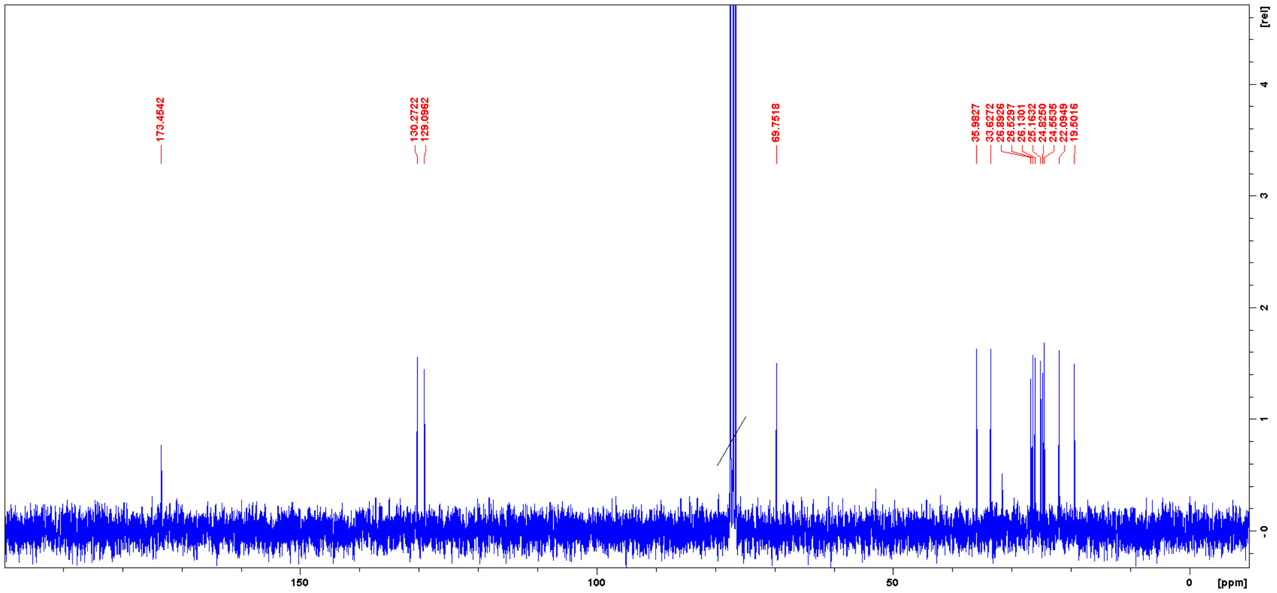

Figure S11: ^{13}C NMR spectrum of (*Z*)-9-tetradecen-13-olide (**2**).

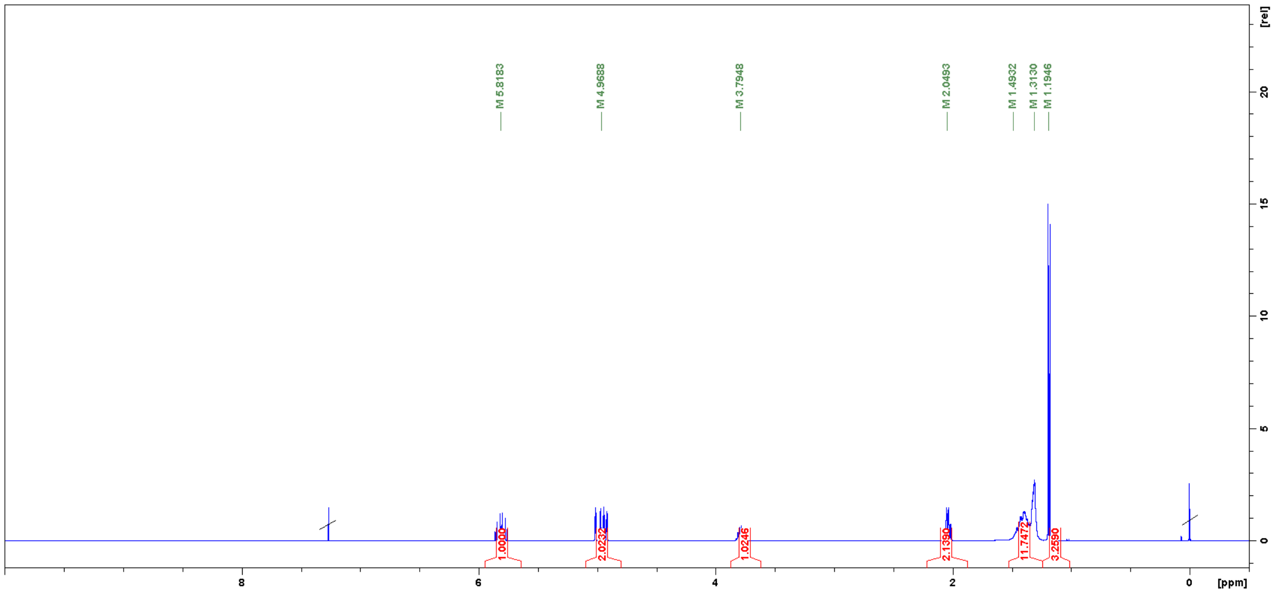

Figure S12: ^{1}H NMR spectrum of dec-9-en-2-ol (**15**).

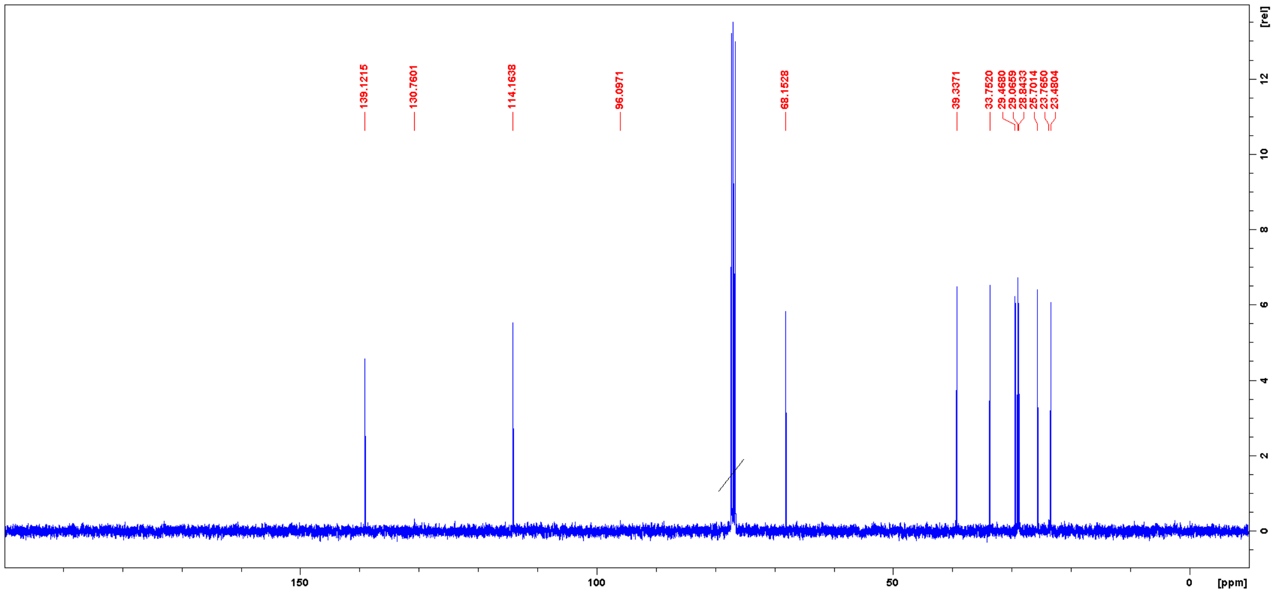

Figure S13: ^{13}C NMR spectrum of dec-9-en-2-ol (**15**).

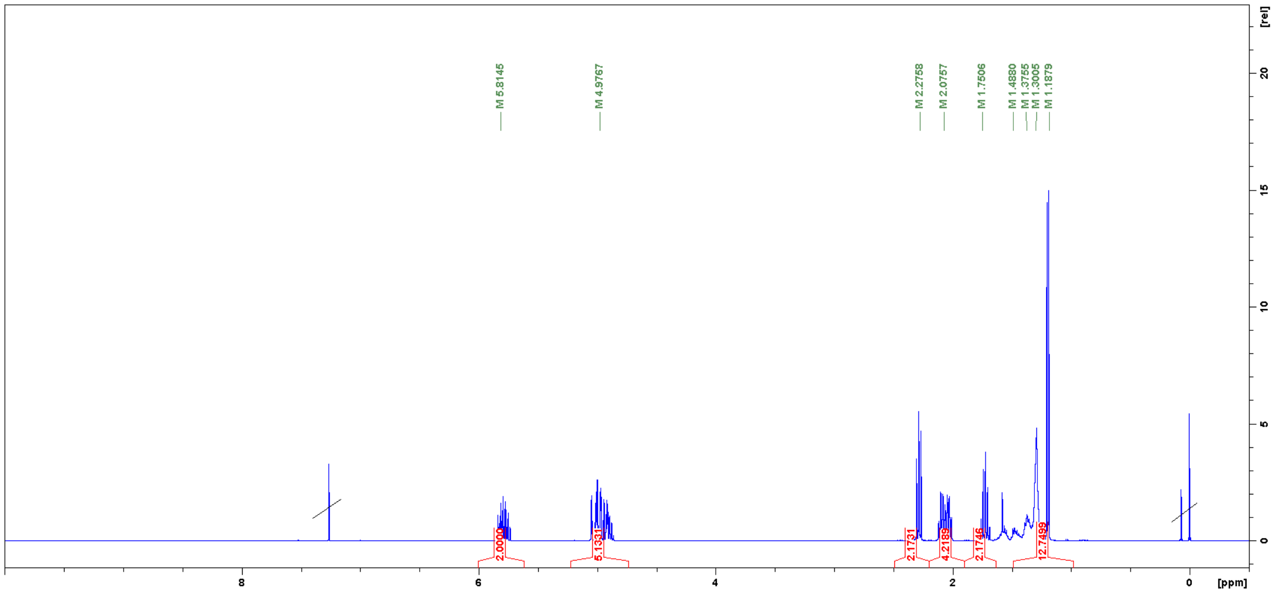

Figure S14: ^{1}H NMR spectrum of dec-9-en-2-yl hex-5-enoate (**17**).

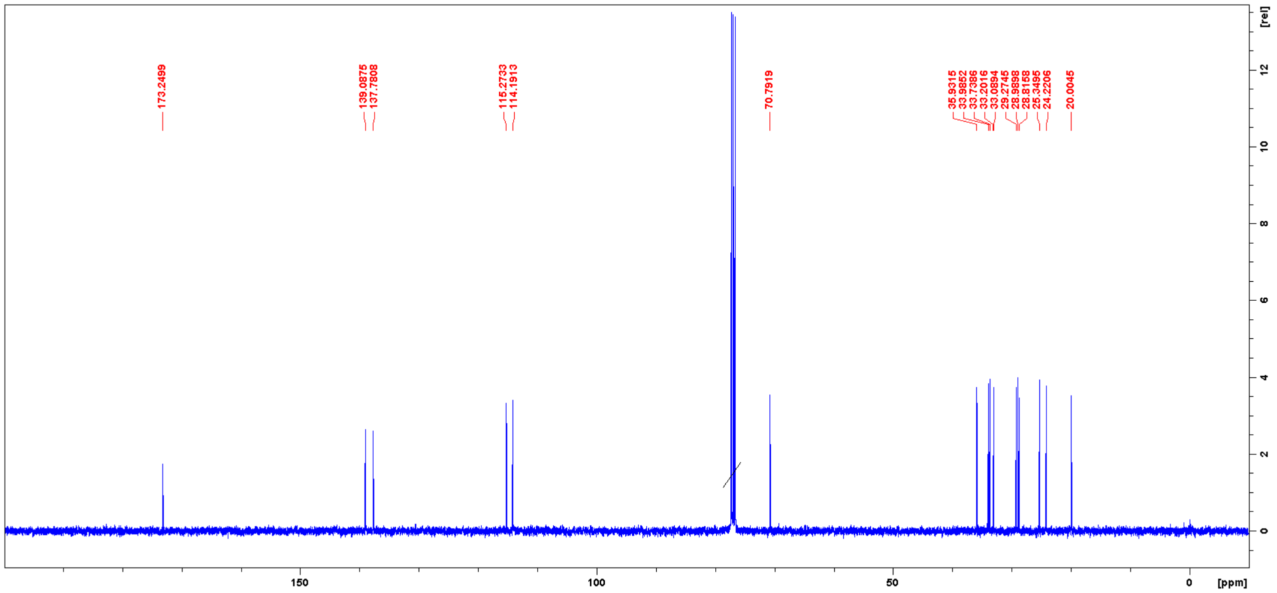

Figure S15: ^{13}C NMR spectrum of dec-9-en-2-yl hex-5-enoate (**17**).

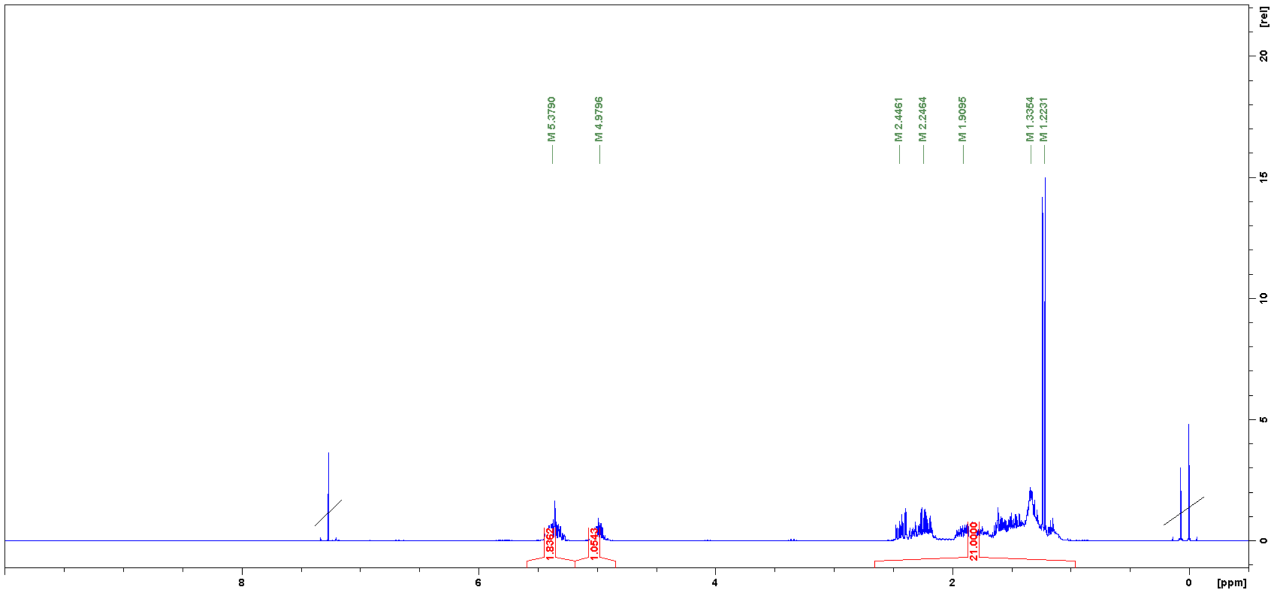

Figure S16: ^{1}H NMR spectrum of (*Z*)-5-tetradecen-13-olide (**1**).

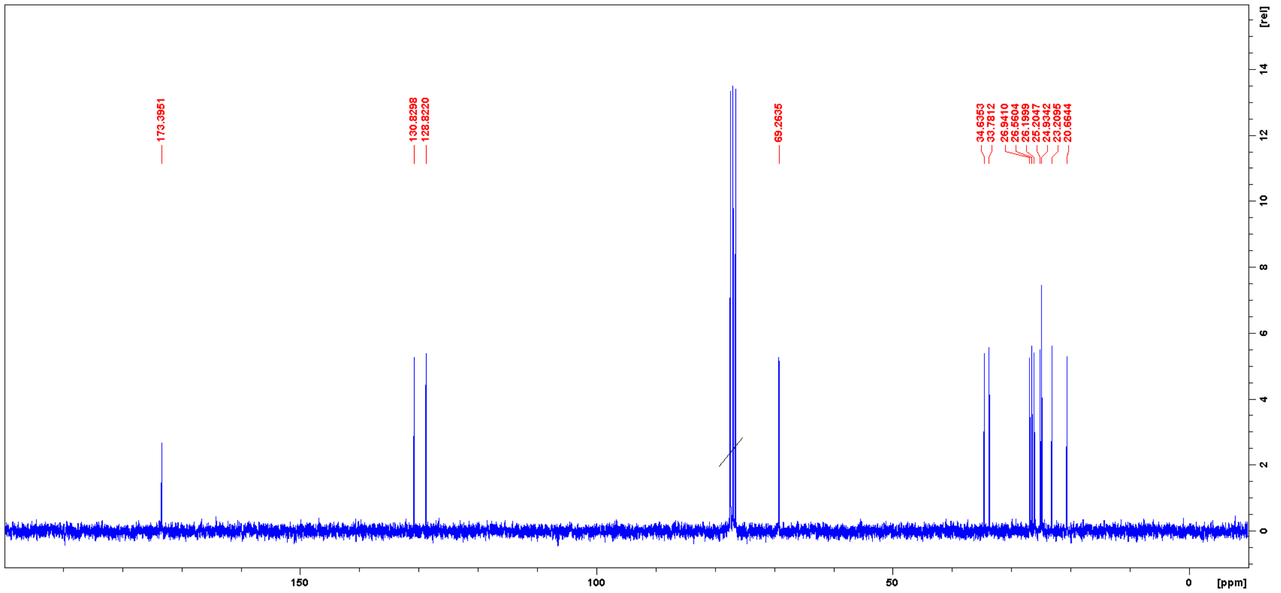

Figure S17: ^{13}C NMR spectrum of (*Z*)-5-tetradecen-13-olide (**1**).

References

S1. Herbert, M. B.; Grubbs, R. H. *Angew. Chem. Int. Ed.* **2015,** *54*, 5018–5024. doi:10.1002/anie.201411588

S2. Hong, S. H.; Sanders, D. P.; Lee, C. W.; Grubbs, R. H. *J. Am. Chem. Soc.* **2005,** *127*, 17160–17161. doi:10.1021/ja052939w

S3. Marx, V. M.; Herbert, M. B.; Keitz, B. K.; Grubbs, R. H. *J. Am. Chem. Soc.* **2013,** *135*, 94–97. doi:10.1021/ja311241q

S4. Rost, D.; Porta, M.; Gessler, S.; Blechert, S. *Tetrahedron Lett.* **2008,** *49*, 5968–5971. doi:10.1016/j.tetlet.2008.07.161

S5. Breinbauer, R.; Jacobsen, E. N. *Angew. Chem. Int. Ed.* **2000,** *2000*, 3604–3607.

S6. Schaus, S. E.; Brandes, B. D.; Larrow, J. F.; Tokunaga, M.; Hansen, K. B.; Gould, A. E.; Furrow, M. E.; Jacobsen, E. N. *J. Am. Chem. Soc.* **2002,** *124*, 1307–1315. doi:10.1021/ja016737l

S7. Searles, S.; Kenneth, J.; Lutz, E. F. *J. Am. Chem. Soc.* **1957,** *1957*, 948–951.

S8. Fantin, G.; Fogagnolo, M.; Giovannini, P. P.; Medici, A.; Pedrini, P. *Tetrahedron Asymmetry* **1995,** *6*, 3047–3053.

S9. Neises, B.; Steglich, W. *Angew. Chem. Int. Ed.* **1978,** *1978*, 522–524.

S10. Noguez, J. H.; Conner, E. S.; Zhou, Y.; Ciche, T. A.; Ragains, J. R.; Butcher, R. A. *ACS Chem. Biol.* **2012,** *7*, 961–966. doi:10.1021/cb300056q

S11. Hamada, T.; Daikai, K.; Irie, R.; Katsuki, T. *Tetrahedron Asymmetry* **1995,** *6*, 2441–2451.
